# Cholesterol-Lowering Probiotics as Potential Biotherapeutics for Metabolic Diseases

**DOI:** 10.1155/2012/902917

**Published:** 2012-05-03

**Authors:** Manoj Kumar, Ravinder Nagpal, Rajesh Kumar, R. Hemalatha, Vinod Verma, Ashok Kumar, Chaitali Chakraborty, Birbal Singh, Francesco Marotta, Shalini Jain, Hariom Yadav

**Affiliations:** ^1^Department of Microbiology & Immunology, National Institute of Nutrition, Hyderabad 50007, India; ^2^Shaheed Udham Singh College of Research & Technology, Punjab, Mohali, Radaur, Haryana, India; ^3^Research and Development Unit, National Heart Centre, Singapore 1687521; ^4^Department of Zoology, M.L.K. Post-Graduate College, Balrampur 271201, India; ^5^Department of Biotechnology, ITS Paramedical College, Ghaziabad 201206, India; ^6^Indian Veterinary Research Institute, Regional Station, Palampur 176061, India; ^7^Hepato-Gastroenterology Unit, S. Giuseppe Hospital, Vittore, 20123 Milano, Italy; ^8^Laboratory of Bioorganic Chemistry, National Institute of Diabetes and Digestive and Kidney Diseases, National Institutes of Health, Bethesda, MD 20892, USA; ^9^Endocrinology, Diabetes, and Obesity Branch, National Institute of Diabetes and Digestive and Kidney Diseases, National Institutes of Health, Bethesda, MD 20892, USA

## Abstract

Cardiovascular diseases are one of the major causes of deaths in adults in the western world. Elevated levels of certain blood lipids have been reported to be the principal cause of cardiovascular disease and other disabilities in developed countries. Several animal and clinical trials have shown a positive association between cholesterol levels and the risks of coronary heart disease. Current dietary strategies for the prevention of cardiovascular disease advocate adherence to low-fat/low-saturated-fat diets. Although there is no doubt that, in experimental conditions, low-fat diets offer an effective means of reducing blood cholesterol concentrations on a population basis, these appear to be less effective, largely due to poor compliance, attributed to low palatability and acceptability of these diets to the consumers. Due to the low consumer compliance, attempts have been made to identify other dietary components that can reduce blood cholesterol levels. Supplementation of diet with fermented dairy products or lactic acid bacteria containing dairy products has shown the potential to reduce serum cholesterol levels. Various approaches have been used to alleviate this issue, including the use of probiotics, especially *Bifidobacterium* spp. and *Lactobacillus* spp.. Probiotics, the living microorganisms that confer health benefits on the host when administered in adequate amounts, have received much attention on their proclaimed health benefits which include improvement in lactose intolerance, increase in natural resistance to infectious disease in gastrointestinal tract, suppression of cancer, antidiabetic, reduction in serum cholesterol level, and improved digestion. In addition, there are numerous reports on cholesterol removal ability of probiotics and their hypocholesterolemic effects. Several possible mechanisms for cholesterol removal by probiotics are assimilation of cholesterol by growing cells, binding of cholesterol to cellular surface, incorporation of cholesterol into the cellular membrane, deconjugation of bile via bile salt hydrolase, coprecipitation of cholesterol with deconjugated bile, binding action of bile by fibre, and production of short-chain fatty acids by oligosaccharides. The present paper reviews the mechanisms of action of anti-cholesterolemic potential of probiotic microorganisms and probiotic food products, with the aim of lowering the risks of cardiovascular and coronary heart diseases.

## 1. Introduction

Although cholesterol is an important basic block for body tissues, elevated blood cholesterol is a well-known major risk factor for coronary heart diseases [1]. WHO has predicted that, by 2030, cardiovascular diseases will remain the leading causes of death, affecting approximately 23.6 million people around the world [2]. It has been reported that hypercholesterolemia contributes to 45% of heart attacks in Western Europe and 35% of heart attacks in Central and Eastern Europe [3]. The risk of heart attack is three times higher in those with hypercholesterolemia, compared to those who have normal blood lipid profiles. The WHO delineated that unhealthy diets, such as those high in fat, salt, and free sugar and low in complex carbohydrates, fruits, and vegetables, lead to increased risk of cardiovascular diseases [4]. Recent modalities for lowering blood cholesterol levels involve dietary management, behavior modification, regular exercise, and drug therapy [5]. Pharmacological agents that effectively reduce cholesterol levels are available for the treatment of high cholesterol; however, they are expensive and are known to have severe side effects [6]. Lactic acid bacteria (LAB) with active bile salt hydrolase (BSH) or products containing them have been suggested to lower cholesterol levels through interaction with host bile salt metabolism [7]. Lactobacilli with BSH activity have an advantage to survive and colonize the lower small intestine where the enterohepatic cycle takes place, and therefore BSH activity may be considered as an important colonization factor [8]. Sanders [9] proposed the mechanism based on the ability of certain probiotic lactobacilli and bifidobacteria to deconjugate bile acids enzymatically, increasing their rates of excretion. Cholesterol, being a precursor of bile acids, converts its molecules to bile acids replacing those lost during excretion leading to a reduction in serum cholesterol. This mechanism could be operated in the control of serum cholesterol levels by conversion of deconjugated bile acids into secondary bile acids by colonic microbes. The use of such orally applied microorganisms (probiotics) is a major aim of the concept of functional food [10, 11]. Recently, there has been much interest in LAB, especially lactobacilli, due to their beneficial effects in health including anti-cholesterol, antidiabetic, antipathogenic, and anticarcinogenic properties and stimulation of the immune system [10, 12–19]. *Lactobacillus plantarum*, the predominating *Lactobacillus* species on oral and intestinal human mucosa, has shown the ability to survive the passage through the human gastrointestinal tract and to establish itself for at least a shorter time in the intestine after consumption [12, 16, 20].

Lactobacilli are frequently used in products for human consumption and can be found as probiotics in infant foods, cultured milks, and various pharmaceutical preparations [10, 21, 22]. One beneficial effect that has been suggested to result from human consumption of probiotic LAB is a reduction in serum cholesterol levels, as suggested by the results of several human and animal studies [23]. This effect can partially be ascribed to an enzymatic deconjugation of bile acids [24–27]. Deconjugated bile salts are less soluble and less efficiently reabsorbed from the intestinal lumen than their conjugated counterparts, which results in excretion of larger amounts of free bile acids in feces [28, 29]. Also, free bile salts are less efficient in the solubilization and absorption of lipids in the gut [30]. Therefore, the deconjugation of bile acids by LAB bacteria could lead towards a reduction in serum cholesterol either by increasing the demand of cholesterol for *de novo* synthesis of bile acids to replace that lost in feces or by reducing cholesterol solubility and, thereby, absorption of cholesterol throughout the intestinal lumen. Moreover, Gilliland et al. [31] observed a significant relationship between cholesterol assimilation by probiotic lactobacilli and their degree of bile deconjugation. BSH, the enzyme responsible for bile salt deconjugation during enterohepatic circulation, has been detected in several LAB species indigenous to the gastrointestinal tract (Table 1) [7, 28, 32, 33]. It has also been suggested that BSH activity should be a requirement in the selection of probiotic organisms with cholesterol-lowering properties, as non-deconjugating organisms do not appear to be able to remove cholesterol from the culture medium to any significant extent [26]. *Lactobacillus fermentum*, a normal resident of the human gut microflora, has been reported to adhere to the epithelial cells, with a preference for the small intestine [34]. It has also been shown to colonize the intestine after oral administration [35] and produce surface-active components that inhibit the adhesion of uropathogenic bacteria [36, 37] (Table 2).

## 2. Bile

**Figure 1:**
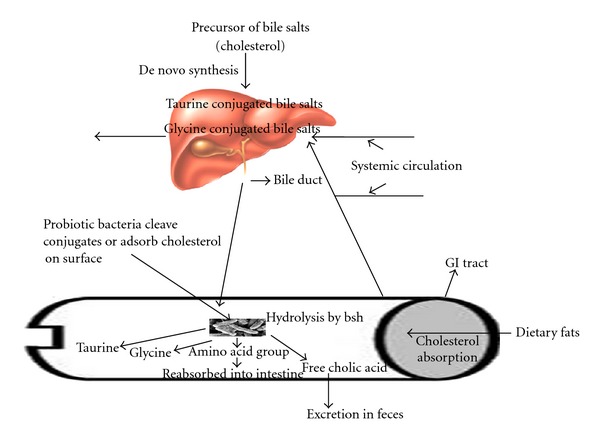
Cholesterol as the precursor for the synthesis of new bile acids and the hypocholesterolemic role of bile salt hydrolase (BSH).

Bile is a yellow-green aqueous solution whose major constituents include bile acids, cholesterol, phospholipids, and the pigment biliverdin [58, 59]. It is synthesized in the pericentral hepatocytes of the liver, stored and concentrated in the gallbladder interdigestively, and released into the duodenum after food intake. Bile functions as a biological detergent that emulsifies and solubilizes lipids, thereby playing an essential role in fat digestion. This detergent property of bile also confers potent antimicrobial activity, primarily through the dissolution of bacterial membranes [60, 61]. Bile acids are saturated, hydroxylated C-24 cyclopentanophenanthrene sterols synthesized from cholesterol in hepatocytes. The two primary bile acids synthesized in the human liver are cholic acid (CA; 3a,7a,12a-trihydroxy-5b-cholan-24-oic acid) and chenodeoxycholic acid (CDCA; 3a,7a-dihydroxy-5b-cholan-24-oic acid). Bile acids are further metabolized by the liver via conjugation (N-acyl amidation) to glycine or taurine, a modification that decreases the Pka to approximately 5. Thus, at physiological pH, conjugated bile acids are almost fully ionized and may be termed bile salts [62]. The primary bile acids, cholic and chenodeoxycholic acid, are synthesized *de novo* in the liver from cholesterol. The solubility of the hydrophobic steroid nucleus is increased by conjugation as an *N*-acyl amidate with either glycine (glycoconjugated) or taurine (tauroconjugated) prior to secretion. The resulting molecules are therefore amphipathic and can solubilize lipids to form mixed micelles. Bile acids are efficiently conserved under normal conditions by a process termed enterohepatic recirculation. Conjugated and unconjugated bile acids are absorbed by passive diffusion along the entire gut and by active transport in the terminal ileum [58]. Reabsorbed bile acids enter the portal bloodstream and are taken up by hepatocytes, reconjugated, and resecreted into bile. Approximately 5% of the total bile acid pool (0.3 to 0.6 g) per day eludes epithelial absorption and may be extensively modified by the indigenous intestinal bacteria [63]. One important transformation is deconjugation, a reaction that must occur before further modifications are possible [64]. Deconjugation is catalyzed by BSH enzymes (EC 3.5.1.24), which hydrolyze the amide bond and liberate the glycine/taurine moiety from the steroid core (Figure 1). The resulting acids are termed unconjugated or deconjugated bile acids.

### 2.1. Identification of bsh Homologs in Probiotic Genomes

The genes that may encode BSH enzymes in the genome sequences of potential probiotic bacteria are available in public databases (National Center for Biotechnology Information genome site (http://www.ncbi.nlm.nih.gov/) and the Joint Genome Institute microbial genomics site (http://genome.jgi-psf.org/)). Several strains (e.g., *Lactobacillus plantarum *WCFS1) possess more than one BSH homolog, which are not identical. The genetic geography of *bsh *regions is not the same in all strains, and in cases where more than one is present they are not located in the same region of the chromosome.

### 2.2. bsh Genes in Probiotic Bacteria

Since variability in *bsh* phenotypes has been observed within isolates of some species [38, 42, 65, 66], it has been speculated that *bsh *genes may have been acquired horizontally [65]. Comparison of the *bsh* gene and surrounding sequences of *L. acidophilus *strain KS-13 and *L. johnsonii *100-100 by Elkins et al. [65] has revealed little or no synteny flanking this locus. It was also noted that *L. johnsonii *100-100 encodes a group II intron protein (maturase *mat*) downstream of *bsh*. In addition to reverse transcriptase activity, these proteins can function as maturases and endonucleases and facilitate movement and splicing of cDNA into the genome. Group II intron proteins are often inserted in or associated with mobile genetic elements [67]. Sequencing of the entire genome of *L. acidophilus *NCFM has revealed that this strain possesses two *bsh *genes (*bshA *and *bshB*). The predicted sequence of the BSH enzymes encoded by these loci share a higher level of similarity to BSH enzymes from other *Lactobacillus *species than to each other, suggesting that they may have been acquired from different sources [68]. In short, BSH is present in all bifidobacterial strains and lactobacilli strains associated with the gastrointestinal environment, but *bsh *genes can potentially be acquired from these strains by other intestinal microorganisms (e.g., *L. monocytogenes*).

## 3. Functions of BSH

The precise function(s) of microbial BSHs is currently not understood, although several hypotheses have been proposed, as follows.

### 3.1. Nutritional Role

The amino acids liberated from bile salt deconjugation could potentially be used as carbon, nitrogen, and energy sources, since glycine may be metabolized to ammonia and carbon dioxide, and taurine may be metabolized to ammonia, carbon dioxide, and sulfate. Bile salt deconjugation may therefore confer a nutritional advantage on hydrolytic strains. In support of this hypothesis, Huijghebaert et al. [69] and Van Eldere et al. [70] observed that certain BSH-positive strains of *Clostridium *utilized the released taurine as an electron acceptor, and growth rates were improved in the presence of taurine and taurine-conjugated bile salts. It has also been noted that transcription of the *Bifidobacterium longum bsh *gene is coupled to a homolog of *glnE *that encodes a glutamine synthetase adenyltransferase that forms part of the nitrogen regulation cascade [39]. However, experiments performed by Tannock et al. [71] and Gilliland and Speck [72] refute this hypothesis since these authors observed that the lactobacilli used in their studies did not utilize the steroid moiety of the bile salt for cellular precursors since neither ring cleavage nor subsequent metabolism occurred. 

#### 3.1.1. Alteration of Membrane Characteristic

The bacteriolytic enzymes lysozyme and phospholipase A2, and antimicrobial peptides such as *α*-defensins, are important contributors to innate immunity in the intestine. The composition, fluidity, permeability, hydrophobicity, and net charge of bacterial membranes all determine the extent of damage by these host defenses. It has been proposed that BSHs facilitate incorporation of cholesterol or bile into bacterial membranes [73–75]. This incorporation may increase the tensile strength of the membranes [76] or may change their fluidity or charge. Cell surface modifications that may result from BSH activity could potentially offer protection against perturbation of the structure and integrity of bacterial membranes by the immune system, and such resistance mechanisms may be important in establishing persistent infections. Such a function may strongly select for commensals possessing BSH enzymes while mitigating against BSH-negative pathogens or other transients.

#### 3.1.2. Bile Detoxification

Studies by various research groups using wild-type and *bsh *mutant pairs have provided a link between bile salt hydrolysis and bile tolerance. A *Lactobacillus amylovorus *mutant with a partial decrease in BSH activity isolated using an *N*-methyl-*N*1-nitro-*N*-nitrosoguanidine mutagenesis strategy displayed decreased growth rates in the presence of bile salts [40]. Also, mutation of *bsh *in *Lactobacillus plantarum *[8] and *Listeria monocytogenes *[60, 61] renders cells significantly more sensitive to bile and bile salts. The precise mechanism by which BSH enzymes play a role in the tolerance of bile is not yet fully understood. However, it has been proposed that since the protonated (non-dissociated) form of bile salts may exhibit toxicity through intracellular acidification in a manner similar to organic acids, BSH-positive cells may protect themselves through the formation of the weaker unconjugated counterparts [77]. This could help negate the drop in pH by recapturing and exporting the cotransported proton. The ratio of glycoconjugated to tauroconjugated bile salts in human bile is usually 3 : 1. *In vitro* experiments have revealed that whereas tauroconjugated bile salts usually only have slight affects (if any) on bacterial cells at every pH examined, glycoconjugated bile salts are extremely toxic at acidic pHs, and *bsh *mutants are significantly more inhibited than corresponding parent cells [60, 61, 77]. Therefore, it has been suggested that BSHs are particularly important in combating the toxic effects of glycoconjugated bile salts at low pH, and BSH activity may be of particular importance at the point where bile enters the duodenum and where acid reflux may occur from the stomach or in localized microenvironments in the intestine when the pH is lowered by lactic acid bacteria. The fact that BSHs have been shown to preferentially hydrolyze glycoconjugated bile salts [78, 79], together with the observation that BSHs have slightly acidic pH optima (usually between pH 5 and 6) [42, 80], may serve to substantiate this theory.

#### 3.1.3. Gastrointestinal Persistence

Since BSHs may combat the deleterious effects of bile (and perhaps components of the innate immune system such as the defensins through cell surface modifications), a role for these enzymes in survival/persistence of strains within the gastrointestinal tract is conceivable. Bateup et al. [81] compared the abilities of three *Lactobacillus *strains which demonstrated various degrees of BSH activity *in vitro* (one strain demonstrated high activity, one showed moderate activity, and one lacked activity) to colonize *Lactobacillus*-free mice. Enumeration of lactobacilli in the gastrointestinal organs 2 weeks after inoculation revealed that all strains colonized equally well, leading to the conclusion that BSH is not essential for colonization. However, a more recent study by Dussurget et al. [82] convincingly demonstrates that BSH contributes to persistence of *L. monocytogenes* within the gastrointestinal tract. A *bsh *mutant demonstrated reduced bacterial fecal carriage after oral infection of guinea pigs (counts of the mutant were 4 to 5 logs lower than the parent after 48 h). It was also observed that intestinal multiplication of the parent could be increased approximately 10-fold by supplying cells with an extra copy of the gene on a plasmid, further confirming the importance of BSH to intestinal persistence [82]. Two obvious differences between this *L. monocytogenes* study and the earlier one of Bateup et al. [81] may account for their different conclusions. First, isogenic *L. monocytogenes *wild-type and *bsh *mutant strains were compared, and it is possible that intrinsic differences between the strains of lactobacilli used in the other study masked the contribution of BSH to intestinal survival. Furthermore, Bateup et al. [81] used *Lactobacillus*-free mice, and it is possible that a role for BSH would be uncovered in a more competitive environment. Therefore, future investigations with bifidobacterial and *Lactobacillus bsh* mutants would be necessary to unequivocally determine whether gastrointestinal persistence is a universal function of BSHs.

### 3.2. Impact of Microbial BSH Activity on the Host

#### 3.2.1. Cholesterol Lowering

**Figure 2:**
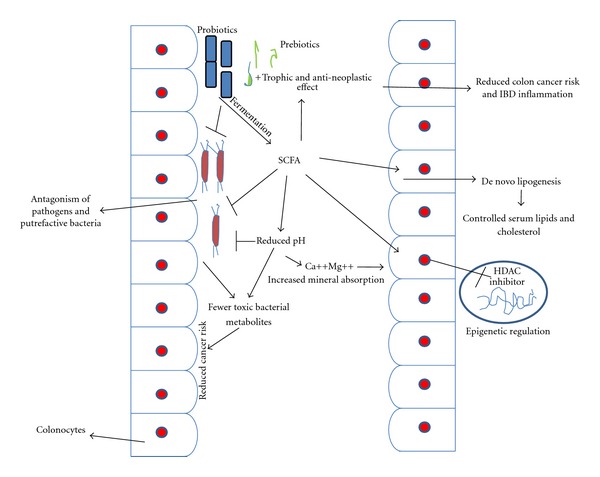
Role of probiotics' metabolites as epigenetic approach to control high cholesterol and colon cancer.

Hypercholesterolemia (elevated blood cholesterol levels) is considered a major risk factor for the development of coronary heart disease, and although pharmacologic agents are available to treat this condition (e.g., statins or bile acid sequestrants), they are often suboptimal and expensive and can have unwanted side effects [83]. Oral administration of probiotics has been shown to significantly reduce cholesterol levels by as much as 22 to 33% [7, 23] or prevent elevated cholesterol levels in mice fed a fat-enriched diet [84]. These cholesterol-lowering effects can be partially ascribed to BSH activity (other possible mechanisms include assimilation of cholesterol by the bacteria, binding of cholesterol to the bacterial cell walls, or physiological actions of the end products of short-chain fatty acid fermentation (Figure 2)) [80]. Deconjugated bile salts are less efficiently reabsorbed than their conjugated counterparts, which results in the excretion of larger amounts of free bile acids in feces. Also, free bile salts are less efficient in the solubilization and absorption of lipids in the gut. Therefore, deconjugation of bile salts could lead to a reduction in serum cholesterol either by increasing the demand for cholesterol for *de novo* synthesis of bile acids to replace those lost in feces or by reducing cholesterol solubility and thereby absorption of cholesterol through the intestinal lumen.

**Impaired Digestive Functions**

Since unconjugated bile acids are less efficient than conjugated molecules in the emulsification of dietary lipids and the formation of micelles, BSH activity may compromise normal lipid digestion and the absorption of fatty acids and monoglycerides could be impaired [28]. Microbial BSH activity has been related to growth defects in chickens [85] but not in mice [81].

## 4. Effects of Probiotics on Plasma Lipids

The idea about the health advantages of fermented milk products in humans goes back to the early 19th century, when it was proposed by Metchnikov that fermenting milks by lactic acid bacteria “prevented intestinal putrefaction” and “helped maintain the forces of the body” [22, 86]. A study on Maasai tribesmen in Africa who have low serum cholesterol showed that they rarely experience coronary heart diseases, despite eating a great deal of meat. They regularly consumed 4-5 liters of fermented whole milk per day. This provided the motivation for investigating fermented milk's possible influence on blood cholesterol [45]. Later, in a study by Mann [46], on twenty-six volunteers, it was found that large amount of yoghurt reduced cholesterolemia, which could be due to a factor in yoghurt that prevents production of cholesterol from acetate. This factor may be either orotic acid or 3-hydroxy-3-methylglutaric acid plus thermophilus milk or methanol soluble of thermophilus milk. Gilliland et al. [31] showed that some strains of *Lactobacillus acidophilus* make it possible for cholesterol to be bound to intestine's lumen and as a result decrease its absorption. Tahri et al. [25] investigated assimilation of cholesterol by *Bifidobacterium *strains and observed that the removal of cholesterol from the growth medium is caused by both bacterial activity and precipitation of cholesterol. Lin and Chen [47] investigated cholesterol-reducing abilities of *L. acidophilus *and found that hypocholesterolemic ability is because of the assimilation of cholesterol by *L. acidophilus* cells or its attachment to the surface of *L. acidophilus *cells. Grunewald [48] observed a strong reduction in serum cholesterol of probiotic fermented milk-fed rats, indicating that cholesterol level in serum can be reduced by consumption of probiotics. A different study conducted on mice with high cholesterol demonstrated that *L. reuteri* was able to reduce blood triglyceride by 38% and cholesterol by 40% and raised the HDL/LDL cholesterol ratio by 20% [49]. Xiao et al. [50] also observed that consumption of *Bifidobacterium *milk leads to a meaningful reduction in triglyceride, low-density lipid, and total cholesterol. Similar results were observed by Abd El-Gawad et al. [51] in a study on rats fed with yoghurt containing *B. lactis *or* B. longum.* Recently, Kumar et al. [52] also explored probiotic *L. plantarum* with potential to control hypercholesterolemia. Lin et al. [53] conducted human studies and observed that blood cholesterol was reduced significantly in volunteers who were given tablets of *L. bulgaricus *and *L. acidophilus *for 16 weeks every day. In another study, hyperlipidemic patients that were given *Lactobacillus sporogenes *for 90 days showed a 35% and 32% decrease in their LDL and total cholesterol levels, respectively [54]. The result by Anderson and Gilliland [87] showed a significant decrease (2.4%) in blood cholesterol for fermented milk containing *L. acidophilus* during controlled clinical trials. In a randomized clinical trial on yoghurt starters plus *Bifidobacterium longum*, participants showed a decrease in total cholesterol [50]. In a randomized, double-blind, placebo-controlled clinical trial, it was demonstrated that *E. faecium *probiotic strain reduced cholesterol levels by 12%. Klein et al. [56] also observed a significant reduction (11.6%) in serum triglyceride levels during the period probiotics consumption in a placebo-controlled, double-blind, randomized crossover study. More recently, Jones et al. [57] reported that yoghurt formulation containing microencapsulated bile salt hydrolase- (BSH-) active *Lactobacillus reuteri* NCIMB 30242 is efficacious and safe for lowering LDL-C, TC, apoB-100, and non-HDL-C in hypercholesterolaemic subjects. Altogether, findings from in vitro systems and animal studies as well as human trails strongly suggest that probiotics have potential to ameliorate the cholesterol metabolic dysfunction, especially mediated through BSH activity and other unknown mechanisms, but the exact mechanism(s) of action for probiotics' mediated decrease in cholesterol levels are not completely known. Here, before discussing the probiotics' mechanism of action on plasma lipids, a summary of lipoprotein synthesis and metabolism is reviewed, as follows for better understanding by general readers the point of actions of probiotics on lipid/cholesterol metabolism.

### 4.1. Plasma Lipoprotein Synthesis and Metabolism

Important organs in the body that are responsible for synthesis and transport of lipoprotein are the liver and the gut. A cystic duct brings bile from the gallbladder to the gut. The liver produces the bile, but it is moved to the gallbladder and remains there to be used. Once a fatty meal arrives at the small intestine, bile salts get into action and help with emulsification of the fats. This makes their digestion and absorption in the gut possible. Fatty acids, triglycerides, and cholesterol combine in the epithelial cells of the gut where they are covered with a layer of protein. These are called chylomicrons [88]. The lymphatic system absorbs these chylomicrons and later releases them into the blood. Chylomicrons find their way to the liver and it turns them into triglyceride and cholesterol. Bile salts do not end up in the gut with the fats. They move down all the way to the ileum, where, most of the bile salts are absorbed once again and entered into the blood. The circulation takes the bile salts back to the liver. They remain in the gallbladder, with bile to be used for the above process again. Some of the bile salts are not absorbed in the small intestine and end up in the colon and are disposed of with feces. The liver makes up for the loss of bile salts by synthesizing them from its cholesterol reservoir. Cells in the liver also synthesize cholesterol and are therefore another major source of the body's cholesterol pool, in addition to the dietary sources of cholesterol. A number of factors, such as genes and diet, modulate the liver to produce cholesterol.

#### 4.1.1. Biosynthesis of Cholesterol

Just less than 50% of the body's cholesterol comes from new biosynthesis, nearly 10% in the liver and 15% in the intestine [88]. Cholesterol synthesis occurs in the microsomes and cytoplasm from the two-carbon acetate group of acetyl-CoA [89]. The biosynthesis of cholesterol goes through the following stages [89]:

- conversion of acetyl-CoAs to 3-hydroxy-3-methylglutaryl-CoA (HMG-CoA),
- conversion of HMG-CoA to mevalonate,
- changing of mevalonate to isopentenyl pyrophosphate,
- changing of isopentenyl pyrophosphate to squalene,
- conversion of squalene to cholesterol.

#### 4.1.2. Regulating Cholesterol Synthesis

In healthy adults, about 1 gram of cholesterol is synthesized and 0.3 gram is consumed per day. The body maintains a relatively constant amount of cholesterol (150–200 mg/dL). This is done mainly through controlling the level of *de novo *synthesis. Dietary intake of cholesterol in part regulates the level of cholesterol synthesis. Both of these cholesterols are then used in the formation of membranes and in the synthesis of the steroid hormones and bile acids [90]. Bile acid synthesis uses most of this cholesterol.

Three separate mechanisms regulate the body's constant supply of cholesterol from cells [88] as follows

- regulation of HMG-CoA reductase (HMGR),
- regulation of extra intracellular free cholesterol via acyl-CoA cholesterol acyltransferase (ACAT),
- regulation of cholesterol levels in plasma via HDL-mediated reverse transport and LDL receptor-mediated uptake,

The cholesterol pool of the liver is used in two important ways. The liver utilizes part of it to produce bile salts, to be stored in the gallbladder as a part of the bile and ends up in the gut. There, the bile salts are involved in the emulsification of fats and their ingestion and absorption. The rest of the cholesterol is used for other requirements of the body. To do this, the liver combines cholesterol from its pool with triglycerides and covers it with a particular protein so that it could be dissolved in the blood. These are somewhat large molecules, known as VLDL (very-low-density lipoproteins). The liver then drains them into the blood. Lipoprotein lipase (LPL) exists in abundance all over the body, especially in the walls of the arteries. This enzyme is involved in removing triglycerides from VLDL cholesterol. In the process, the VLDL shrinks in size and a relatively larger portion of it is made up of what is called intermediate-density lipoproteins, or IDL.

**Low-Density Lipoprotein (LDL)**

As the process continues and more triglycerides are taken away, what is left is a dense molecule referred to as low-density lipoprotein (LDL). This lipoprotein still maintains a large amount of cholesterol. The protein layer allows the tissues to use this cholesterol, LDL receptors on these tissues that make this interaction possible. In the tissues such as those of the liver and the inner layer of the arterial wall, cholesterol is taken away from low-density lipoproteins. Free radicals in the body are very reactive and oxidative compounds that can oxidize low-density lipoprotein cholesterol and help atherosclerotic plaque to form in the arteries. Antioxidants in the body can inhibit this process [91].

**High-Density Lipoprotein (HDL)**

The liver also produces another type of lipoprotein, named high-density lipoprotein. This is different from VLDL, which is also produced in the liver. It has little triglyceride and cholesterol and has a particular protein covering. High-density lipoprotein collects the surplus cholesterol that cholesterol metabolizing cells cannot utilize. Lecithin-cholesterol acyltransferase is an enzyme that is responsible for transporting surplus cholesterol back to HDL molecules. Unused cholesterol from arteries, liver, and other tissues is absorbed by HDL cholesterol. There is evidence that even some oxidized LDL can be removed by the LCAT and HDL cholesterol [92]. As HDL circulates in the body and collects the cholesterol from tissues, it becomes mature and goes back to the liver. There, it is identified by its lipoprotein covering and is lodged in the liver's cholesterol pool.

**Apo-A-1**

Apo-A-1 is the main apolipoprotein in HDL cholesterol and performs a key function of collecting surplus cholesterol from the outer cells and transporting it back to the liver. It also has antioxidant and anti-inflammatory properties [93].

Apo33-B/Apo-A ratio is an indicator of cardiovascular risk. The higher the ratio, the higher the probability of cholesterol deposits in the walls of the arteries [94].

**Apo-B**

Apo-B is found in all of the atherogenic particles; VLDL, IDL, as well as large and small dense LDL cholesterol. They all have one Apo-B molecule inside them. The number of Apo-B, therefore, is an indicator of the number of the above particles. Apo-B helps to capture these particles from the walls of the arteries. On the other hand, the Apo-B formed in the liver helps with stabilization and transfer of cholesterol and triglycerides in plasma IDL, VLDL, and sd-LDL, and with the collecting of cholesterol in the liver and the outer tissues. Of all the Apo-B particles in the blood, over ninety percent are in low-density-lipid cholesterol. Low-to-normal LDL cholesterol may indicate an increase in highly atherogenic sd-LDL particles that are readily oxidized, leading to increased formation of plaques on the arteries walls. Apo-B/Apo-A ratio is an indicator of cardiovascular risk. The higher the ratio, the higher the probability of cholesterol deposits in the walls of the arteries [94].

### 4.2. Probiotics' Mechanism of Action on Lipids

It has been proposed that, when probiotics settle in the gut, they ferment indigestible carbohydrate from food. Their action raises the short-chain fatty acids (SCFAs) in the gut [86]. SCFAs are produced from peptide, polysaccharide, protein, and oligosaccharide, mainly by anaerobic bacteria, and are the final product of bacteria's activity into the GI tract. In terms of quantity, carbohydrates are the main source of short-chain fatty acids [86]. These large molecules get depolymerised by a variety of hydrolytic enzymes that are produced by bacteria and allow the organisms to ferment their sugar content. SCFA can lower the lipids in blood through blocking synthesis of hepatic cholesterol and/or through redirecting plasma cholesterol toward the liver [95]. A hundred to 450 mmol of the SFCA is produced in the large intestine every day with relative proportions of acetate, propionate, and butyrate being about 60 : 20 : 15 depending on the substrate [86]. While acetate seems to increase total cholesterol, propionate increases glucose in the blood and reduces hypercholesterolemia response caused by acetate. Propionate does that by decreasing its use by the liver, for cholesterol and fatty acids synthesis. In addition, SCFAs are potential modulator of food intake and energy sensing process into the brain, which might indirectly play an important role in reduction of cholesterol and other metabolism deranging lipids into the host body [96]. Micelles, which play a role in the absorption of cholesterol in the intestine, are produced by bile salts, cholesterol, and phospholipids. By producing bile acids through deconjugating the bile salts in the small intestine, probiotics prevent micelle production. When cholesterol enters the enterohepatic circulation, it is death in the same way. Probiotics by using hydroxysteroid dehydrogenase, and conjugated bile acid hydrolase enzymes, breakdown the bile acid and hydrolyze bile salts. By doing so bile acids' enterohepatic circulation will be disrupted [97–99]. Hydroxymethylglutarate CoA (HMG CoA) is another compound that helps probiotics block HMG-CoA reductase activity, which is a rate-limiting enzyme and is involved in endogenous production of cholesterol. Probiotic bacteria reduce absorption of cholesterol in the intestine by binding and hence incorporating it to the cell membrane. Cholesterol can also be assimilated during growth [100]. All of the above-mentioned activities together help with the cholesterol-lowering actions of probiotics.

### 4.3. Mechanisms of Cholesterol-Lowering Effects

Past *in vitro *studies have evaluated a number of mechanisms proposed for the cholesterol-lowering effects of probiotics and prebiotics. One of the purported mechanisms includes enzymatic deconjugation of bile acids by bile salt hydrolase of probiotics. Bile, a water-soluble end product of cholesterol in the liver, is stored and concentrated in the gallbladder and released into the duodenum upon ingestion of food [101]. It consists of cholesterol, phospholipids, conjugated bile acids, bile pigments and electrolytes. Once deconjugated, bile acids are less soluble and absorbed by the intestines, leading to their elimination in the feces. Cholesterol is used to synthesize new bile acids in a homeostatic response, resulting in lowering of serum cholesterol [101] (Figure 1). In an *in vitro *study, Jones et al. [102] evaluated the role of bile salt hydrolase in cholesterol lowering using *Lactobacillus plantarum*. The authors found that BSH activity was able to hydrolyze conjugated glycodeoxycholic acid and taurodeoxycholic acid, leading to the deconjugation of glyco- and taurobile acids. The hypocholesterolemic effect of the probiotics has also been attributed to their ability to bind cholesterol in the small intestines. Usman [27] previously reported that strains of *Lactobacillus gasseri *could remove cholesterol from laboratory media via binding onto cellular surfaces. The ability of cholesterol binding appeared to be growth and strain specific. Kimoto et al. [103] later strengthened such a hypothesis by evaluating the removal of cholesterol by probiotics cells during different growth conditions. Live and growing cells were compared to those that were nongrowing (live but suspended in phosphate buffer) and dead (heat-killed). It was observed that, although growing cells removed more cholesterol than dead cells, the heat-killed cells could still remove cholesterol from media, indicating that some cholesterol was bound to the cellular surface. Cholesterol was also removed by probiotics by incorporation into the cellular membranes during growth. Kimoto et al. [103] have examined the removal of cholesterol by several strains of lactococci from media. A difference in the fatty acid distribution pattern was observed for cells grown in the presence and absence of cholesterol. Lipids of probiotics are predominantly found in the membrane, suggesting that cholesterol incorporated into the cellular membrane had altered the fatty acid composition of the cells. The incorporation of cholesterol into the cellular membrane increased the concentration of saturated and unsaturated fatty acids, leading to increased membrane strength and subsequently higher cellular resistance toward lysis [104, 105]. Lye et al. [104] also further evaluated this mechanism by determining the possible locations of the incorporated cholesterol within the membrane phospholipid bilayer of probiotic cells. Fluorescence probes were incorporated into the membrane bilayer of probiotic cells that were grown in the absence and presence of cholesterol. Enrichment of cholesterol was found in the regions of the phospholipid tails, upper phospholipids, and polar heads of the cellular membrane phospholipid bilayer in cells that were grown in the presence of cholesterol compared to the control cells, indicating incorporation of cholesterol in those regions. Cholesterol can also be converted in the intestines to coprostanol, which is directly excreted in feces. This decreases the amount of cholesterol being absorbed, leading to a reduced concentration in the physiological cholesterol pool. Possible conversion of cholesterol into coprostanol by bacteria has been evaluated by Chiang et al. [106]. In their study, it was found that cholesterol dehydrogenase/isomerase produced by bacteria such as *Sterolibacterium denitrificans *was responsible for catalyzing the transformation of cholesterol to cholest-4-en-3-one, an intermediate cofactor in the conversion of cholesterol to coprostanol. This served as a fundamental for further evaluations using strains of probiotic bacteria. In a recent *in vitro *study, Lye et al. [105] evaluated the conversion of cholesterol to coprostanol by strains of lactobacilli such as *Lactobacillus acidophilus, L. bulgaricus,* and *L. casei *ATCC 393 via fluorometric assays. The authors detected both intracellular and extracellular cholesterol reductase in all strains of probiotics examined, indicating possible intracellular and extracellular conversion of cholesterol to coprostanol. The concentration of cholesterol in the medium also decreased upon fermentation by probiotics accompanied by increased concentrations of coprostanol. This mechanism warrants further evaluations as cholesterol reductase is also directly administered to humans to convert cholesterol to coprostanol in the small intestines for a bloodstream cholesterol-lowering effect. Most of the hypotheses raised to date are based on *in vitro *experiments, and few attempts have been made to evaluate the possible hypocholesterolemic mechanisms based on *in vivo *trials. Most of the *in vivo *trials conducted thus far have focused heavily on verifying the hypocholesterolemic effects of probiotics, rather than the mechanisms involved. Liong et al. [107] had evaluated the hypocholesterolemic effect of a synbiotic and the possible mechanisms involved by using hypercholesterolemic pigs. In their parallel 8-week study, the authors found that the administration of a synbiotic containing *L. acidophilus *ATCC 4962, fructooligosaccharides, inulin, and mannitol decreased plasma total cholesterol, LDL-cholesterol, and triacylglycerols compared to the control. These lipoproteins were subsequently subfractionated and characterized. Pigs supplemented with the synbiotic had a lower concentration of cholesteryl esters in the LDL particles, accompanied by a higher concentration of triacylglycerol. Triacylglycerol-enriched LDL particles are more susceptible to hydrolysis and removal from blood, while loss of cholesteryl esters forms smaller and denser LDL particles leading to a higher removal from blood compared to larger LDL particles. The authors also found that the administration of the synbiotic led to higher concentration of cholesteryl esters in the HDL particles. HDL is termed as the beneficial cholesterol attributed to its role of transporting cholesterol to the liver for further hydrolysis. Cholesterol is transported as cholesteryl esters in the core of HDL. Thus, it was suggested that the synbiotic induced a hypocholesterolemic effect via altering the pathways of cholesteryl esters and lipoprotein transporters. Prebiotics such as inulin and fructooligosaccharides are soluble, indigestible, viscous, and fermentable compounds that contribute to hypocholesterolemia via two mechanisms: decreasing cholesterol absorption accompanied by enhanced cholesterol excretion via feces and the production of short-chain fatty acids (SCFAs) upon selective fermentation by intestinal bacterial microflora (Figure 2) [108]. Using hypercholesterolemic-induced rats, Kim and Shin [109] also found that the administration of inulin for 4-weeks decreased serum LDL-cholesterol with increased serum HDL-cholesterol levels (*P* < 0.05) compared to the control. Rats fed with inulin also showed higher excretions of fecal lipid and cholesterol compared to the control (*P* < 0.05), mainly attributed to reduced cholesterol absorption. Similar to indigestible fibers, soluble indigestible prebiotics have been postulated to increase the viscosity of the digestive tract and increase the thickness of the unstirred layer in the small intestine and thus inhibiting the uptake of cholesterol [110]. This may have led to a higher cholesterol catabolism in the liver that contributed to a hypocholesterolemic effect.

## 5. Conclusion and Future Prospects

Probiotics have received much attention on their proclaimed health benefits which include improvement in lactose intolerance, increase in natural resistance to infectious disease in gastrointestinal tract, suppression of cancer, reduction in serum cholesterol level, and improved digestion. In addition, there has been considerable interest in the effect of probiotics on human lipid metabolism, and numerous studies have focused on the potential hypocholesterolemic activity of probiotics in human. Despite these claimed benefits from the human clinical studies carried out for the last two decades, a decisive outcome has failed to be reached due to controversies raised. Also, the exact mechanism for cholesterol removal is poorly understood. Several possible mechanisms for cholesterol removal by probiotics have been proposed including assimilation of cholesterol by growing cells, binding of cholesterol to cellular surface, incorporation of cholesterol into the cellular membrane, deconjugation of bile via bile salt hydrolase, and coprecipitation of cholesterol with deconjugated bile; however, some of these mechanisms are strain dependent, and conditions generated under laboratory conditions would not be practical in the *in vivo *systems. Such discrepancies in the data of different effects on serum cholesterol levels may come from the differences in genus, species, and strains of lactic acid bacteria. Even though the hypocholesterolemic mechanism of probiotics has not yet been fully understood, it is an established fact that cholesterol and bile salt metabolism are closely linked. Recently “BSH hypothesis” has being proposed to explain cholesterol-lowering effects of probiotics. More recently, the hypocholesterolemic effects of some probiotics, which showed high BSH activities from *in vitro *trials, have been confirmed in human as well as in animals. However, the hypocholesterolemic mechanism of probiotics based on the BSH hypothesis has not yet been sufficiently elucidated. Moreover, considering that a number of commercial probiotic strains exhibit high BSH activities, further studies are needed to determine whether the BSH activity of the probiotics strains is beneficial or detrimental to the host. In probiotic research, bile tolerance is considered of primary importance in the selection of strains as bile tolerance enables the bacteria to survive its transit along the duodenum and subsequently to grow and colonize the gut epithelia. Thus, it is important to understand the physiological and molecular mechanisms by which enteric microorganisms including bifidobacteria have evolved to resist against antimicrobial activity of bile in the GI tract. Further investigation on the conserved and variable regions of the *bsh *genes from various species could be useful for the development of alternative phylogenetic marker for bifidobacteria. Furthermore, one of the future challenges will be to unravel the physiological impacts of bile salt hydrolase activity on the enzyme-producing bacterial and mammalian cells.

## Figures and Tables

**Table 1:** List of some potential bacteria showing bile salt hydrolase (BSH) activity.

Probiotic organisms with BSH activity	References
*Bifidobacterium adolescentis*	[38]
*B. animalis*	[38]
*B. breve*	[38]
*B. infantis*	[39]
*B. longum*	[39]
*Bifidobacterium *sp.	[40, 41]
*Lactobacillus acidophilus*	[41–43]
*L. casei*	[41–43]
*L. fermentum*	
*L. gasseri*	[38, 41]
*L. helveticus*	[38]
*L. paracasei *subsp. *paracasei *	[44]
*L. rhamnosus*	[38, 44]
*L. plantarum*	[8, 19]

**Table 2:** Summary of major findings for probiotic mediated cholesterol reduction.

S. No.	Probiotic organism	Experimental system	Major findings	Reference
1	Unknown (fermented milk)	Maasai tribesmen in Africa	Low cholesterol	[45]

2	Unknown (Yogurt)	Human subjects	Reduced cholesterol	[46]

3	*Lactobacillus acidophilus*	Culture media	Cholesterol removal Better survival in cholesterol media	[31]

4	*Bifidobacterium*	Culture media	Removal of cholesterol	[25]

5	*L. acidophilus*	Culture media	Cholesterol assimilation	[47]

6	Probiotic fermented milk	Rats	Cholesterol reducing efficacy	[48]

7	*L. reuteri*	Mice	Reduced blood cholesterol Decreased triglycerides	[49]

8	*Bifidobacterium *milk	Rats, Human	Reduced cholesterol Decreased triglyceride Decreased LDL Increased HDL	[50]

9	Yoghurt containing *B. lactis *or* B. longum *	Rats	Reduced cholesterol Decreased triglyceride Decreased LDL Increased HDL	[51]

10	*L. plantarum*	Culture media	Cholesterol assimilation	[52]

11	*L. bulgaricus *and *L. acidophilus *	Human	Decreased cholesterol	Lin et al. [53]

12	*Lactobacillus* *sporogenes *	Human	Decreased Cholesterol Reduced LDL-cholesterol	[54].

13	*L. acidophilus*	Human	Decreased cholesterol	Gilliland [55]

14	*E. faecium*	Human	Decreased cholesterol levels Decreased triglyceride Decreased LDL Increased HDL	[56]

15	Microencapsulated bile salt hydrolase- (BSH-) active *Lactobacillus reuteri* NCIMB 30242	Human	Reduced LDL-cholesterol Decreased total cholesterol Decreased apoB-100 Decreased non-HDL-cholesterol	[57]
