# Corrigendum to “Cholesterol-Lowering Probiotics as Potential Biotherapeutics for Metabolic Diseases”

**DOI:** 10.1155/2022/3952529

**Published:** 2022-04-01

**Authors:** Manoj Kumar, Ravinder Nagpal, Rajesh Kumar, R. Hemalatha, Vinod Verma, Ashok Kumar, Chaitali Chakraborty, Birbal Singh, Francesco Marotta, Shalini Jain, Hariom Yadav

**Affiliations:** ^1^Department of Microbiology & Immunology, National Institute of Nutrition, Hyderabad, India; ^2^Department of Biotechnology, JMIT Institute of Technology, Radaur, Haryana, India; ^3^Research and Development Unit, National Heart Centre, Singapore; ^4^Department of Zoology, M.L.K. Post-Graduate College, Balrampur, India; ^5^Department of Biotechnology, ITS Paramedical College, Ghaziabad, India; ^6^Indian Veterinary Research Institute, Regional Station, Palampur, India; ^7^Hepato-Gastroenterology Unit, S. Giuseppe Hospital, Vittore, Milano, Italy; ^8^National Institute of Diabetes and Digestive and Kidney Diseases, NIH, MD, USA

The article titled “Cholesterol-Lowering Probiotics as Potential Biotherapeutics for Metabolic Diseases” [1] was found to contain material from [2], as raised on PubPeer [5]. The article was also found to contain materials from other published work and to have missing attributions and errors in citations. The articles are as follows:

- Máire Begley, Colin Hill and Cormac G. M. Gahan, “Bile Salt Hydrolase Activity in Probiotics,” doi: 10.1128/AEM.72.3.1729-1738.2006. Appl. Environ. Microbiol. March 2006 vol. 72 no. 3 1729-1738 [2].
- Ooi, L.-G.; Liong, M.-T. Cholesterol-Lowering Effects of Probiotics and Prebiotics: A Review of in Vivo and in Vitro Findings. Int. J. Mol. Sci. 2010, 11, 2499-2522 [3].
- M Ratna Sudha, Prashant Chauhan, Kalpana Dixit, Sekhar Babu, Kaiser Jamil, “Probiotics as complementary therapy for hypercholesterolemia,” Biology and Medicine, 1 (4): Rev4, 2009 [4].

The authors apologize for these errors and the corrected version of the article is shown below:

Cardiovascular diseases (CVD) are among the prime causes of deaths in adults, especially in Western world human populations. High serum lipid levels are the principal causes of CVD and associated disabilities. In vivo studies comprising of model animals and clinical trials have revealed the connection between high cholesterol levels and increased risk of coronary heart disease (CHD). Nutrition strategies are aimed at preventing CVD warrant shifting to the diet that contains low levels of saturated fats. Undoubtedly, in experimental conditions, such as low-fat diet, it offers effective means of minimizing blood cholesterol levels on a population basis; these appear to be less effective in practice, primarily due to poor compliance owing to less taste and acceptance of the consumer. Due to the low consumer compliance, attempts are made to evolve alternative diets that assist in reducing the blood cholesterol levels. Fermented foods containing lactic acid bacteria (LAB) can possibly lessen the serum cholesterol and the risks associated with hypercholesterolemia. Probiotics have received considerable attention in view of their proclaimed health benefits, such as improved lactose utilization, resistance to gastrointestinal (GI) infections, suppression of inflammatory diseases and cancer, antidiabetic effects, and minimizing serum cholesterol. The possible mechanisms underlying cholesterol-lowering effects of probiotics include the assimilation and incorporation of cholesterol into dividing somatic cells of the host, deconjugation of bile salts via bile salt hydrolase (BSH) (EC 3.5.1.24), coprecipitation of cholesterol with deconjugated bile, and dietary fiber-mediated binding of the bile salts. This review discusses the mechanisms of actions of hypocholesterolemic activities of probiotics as well as the fermented foods that contain probiotics, thereby ultimately lowering the risk of cholesterol-mediated CHD and CVD.

## 1. Introduction

Although cholesterol is an important constituent of human cells, abnormally elevated levels of cholesterol in blood vascular or circulatory system are a potential causative factor of CHD [1]. According to World Health Organization (WHO) predictions, by 2030, the CVDs will spread globally and account for the majority of the deaths, affecting around 23.6 million people worldwide [2]. According to Yusuf et al. [3], the hypercholesterolemia is responsible to 45% incidences of cardiac attacks in Western Europe and about 35% incidences of heart attacks and associated diseases in Central and Eastern Europe. Further, patients with hypercholesterolemia have about three times higher risk of heart attack as compared to people with a normal blood lipid profile. Altered food habits and shift towards diets enriched with high saturated and animal-origin fats, salt and readily fermentable sugar, and low fiber, fruits, and vegetables are responsible for increased incidences of CVDs [4]. Dunn-Emke et al. [5] have suggested that nutritional management, altered life style, regular physical exercise, and timely medication could reduce the blood cholesterol. According to Bliznakov [6], the pharmacological interventions that efficiently reduce cholesterol levels are available to treat hypercholesterolemia, i.e., high blood cholesterol; however, the treatment is costly and may have severe adverse effects in some cases.

Nature has blessed humans with microorganisms with remarkable prohealth attributes that have coevolved with host. For instance, lactic acid bacteria (LAB), the normal microflora, possess bile salt hydrolase (BSH) activity that plays pivotal role in diminishing the levels of cholesterol levels through multiple mechanisms [7]. Lactobacilli possessing BSH and antimicrobial activity colonize most mucosal sites such as genitourinary tract and lower parts of the intestine. Therefore, BSH activity might contribute multiple prohealth benefits [8]. Certain probiotic strains of *Lactobacillus* sp. and *Bifidobacterium* sp. produce enzymes to deconjugate bile acids, thereby facilitating their elimination from the body [9]. Cholesterol, due to being a natural precursor of bile acids, is converted to bile acids, hence replaced or eliminated as a part of excretion. Proficiency of the colonic microbes such as LAB to transform deconjugated bile acids into secondary bile acids could be exploited to control serum cholesterol levels. Therefore, application of orally administered probiotics is the key objective of the conception of bioactive or functional foods [10, 11]. Recently, a surge is noted in human interest in LAB, especially lactobacilli, due to their multi prohealth benefits including antidiabetic, anticholesterol, antipathogenic, and anticarcinogenic properties and the immunomodulatory activities [11–20]. Orally administered *Lactobacillus plantarum* was found to endure the passage through the gastrointestinal (GI) tract and establish in human intestine [12, 14, 21]. Notably, the LAB and other probiotic microbial strains are often used in dairy foods such as yoghurt, cultured milks, and infant food pharmaceutical formulations [9, 11, 22].

Several studies on human cohorts as well as animal models have suggested reduction in cholesterol levels in blood serum as one of the major beneficial effects of consuming probiotics [23]. The hypocholesterolemic effect is partly attributed to the gut microbial biocatalytic deconjugation of the bile acids [24–27]. It has been found that, compared to conjugated bile salts, the deconjugated bile salts being less soluble are not readily and efficiently reabsorbed from intestinal lumen and hence are excreted profusely as free bile acids along with feces [28, 29]. Besides, free bile salts have less efficacy to solubilize and absorb the lipids in the intestinal tract [30]. Hence, the ability of certain probiotic LAB strains to deconjugate bile acids thereby reducing the serum cholesterol via mechanisms, such as (a) increased requirement of cholesterol for *de novo* synthesis of bile acids to compensate for that lost in fecal discharge or (b) reduced overall solubility and absorption of cholesterol, could be a promising strategy of probiotics. Gilliland et al. [31] noted a strong correlation between cholesterol assimilation and bile deconjugation abilities of probiotic *L. acidophilus*, showing that certain strains of probiotic *L. acidophilus*, may be helpful in reducing the blood cholesterol levels [31].

BSH, the important enzyme involved in deconjugation of bile salts during enterohepatic circulation, is found in several *Lactobacillus* and other LAB species residing in the gastrointestinal tract (Table 1) [7, 29, 32, 33]. Accordingly, it has been proposed that the BSH activity should also be considered one of the fundamental criteria while selecting and nominating a given bacterial strain as probiotics, particularly given that the microorganisms lacking BSH activity fail to remove the cholesterol [26, 31]. *L. fermentum*, a common human GI tract symbiont is known to adhere to epithelial cells small intestine [34]. Orally administered probiotics inhibit adhesion of uropathogenic bacteria by multiple mechanisms including production of surface-active components [35–37].

## 2. Bile

Bile forms a yellowish-green aqueous solution comprising of bile acids, cholesterol, certain phospholipids, and the pigment known as biliverdin [38, 39]. Bile is synthesized primarily in pericentral hepatocytes and transported to the gallbladder for concentration and storage. The bile is poured into the lumen of duodenum upon stimulation by ingested food. The bile plays an indispensable role in digestion of dietary fats/lipids by functioning like a biological detergent for their emulsification and solubilization. The detergent property of bile helps dissolve bacterial membranes, hence exhibiting a potent antimicrobial activity [40, 41]. Bile acids are produced from cholesterol by a saturation process, hydroxylation at C-24 cyclopentanophenanthrene as sterols. Two primary bile acids, namely, cholic acid (CA; 3a,7a,12a-trihydroxy-5bcholan-24-oic acid) and chenodeoxycholic acid (CDCA; 3a,7a-dihydroxy-5b-cholan-24-oic acid), are synthesized in the liver. Conjugation (N-acyl amidation) of bile acids to glycine or taurine decreases the Pka to about 5 in the human liver. Therefore, the conjugated bile acids, being fully ionized at physiological pH, are typically called bile salts [42]. De novo synthesis of the primary bile acids (CA and CDCA) from cholesterol takes place in the hepatic cells. Conjugation in the form of an *N*-acyl amidate with glycine (glycoconjugated) and/or taurine (tauroconjugated) preceding to secretion increases the solubility of hydrophobic steroid nucleus. The resulting molecules become amphipathic and can help to solubilize lipids and form mixed micelles. Subsequently, the enterohepatic recirculation or circulation of biliary acids, drugs, or other substances from the liver to the bile conserves bile under normal biological conditions. In the upper part of the intestine, the conjugated as well as nonconjugated bile salts are absorbed via passive diffusion, while in the terminal tract or ileum, the bile salts are absorbed by active transport [38]. The absorbed bile acids enter into portal blood circulation and reach the liver and hepatocytes. After reconjugation, the bile salts are secreted into the bile housed in the gall bladder. Every day, about 5% of the bile acid reserve (0.3 to 0.6 g/day) is released into and mixed with semidigested food in the duodenum and modified by the resident gut microbiota [43]. Consequently, the occurrence and completion of deconjugation are indispensable prior to subsequent auxiliary modifications of the bile salts [44]. The deconjugation process is catalyzed by BSH that catalyzes hydrolysis of amide bond and releases the glycine and/or taurine moieties from the core steroid nucleus (Figure 1).

### 2.1. *bsh* Homologs in the Genomes of Probiotics

Genes encoding the BSH enzymes in the genome of various microbes including probiotic strains are already unveiled and available in public domains viz. National Center for Biotechnology Information (NCBI) genome site (http://www.ncbi.nlm.nih.gov/) and Joint Genome Institute Microbial Genomics site (http://genome.jgi-psf.org/). It may be noted, however, that specific bacterial strains (for instance, *L. plantarum* WCFS1) are known to harbor more than one BSH homolog that is not identical. The genetic layout of regions corresponding to the *bsh* genes is not always comparable in all of the microbial species and strains. Additionally, in specific milieus wherein more than one *bsh* gene is present in the genome, the *bsh* genes may be present at different chromosomal loci or regions.

### 2.2. *Bsh* Genes in Probiotic Bacteria

Given that the *bsh* phenotypes varies within the strains of same species and genera [45–49], it is hypothesized that the *bsh* genes in probiotics (like *L. johnsonii* 100-100) may have been acquired horizontally and that the BSH activity is indispensable for probiotics to colonize the GI tract [47]. Besides reverse transcriptase action, these proteins also possess and perform maturase and restriction endonuclease functions thereby catalyzing the movement and splicing of cDNA into the microbial genome. Notably, the group II intron proteins have also been inserted in or linked with some mobile genetic elements [50]. Whole genome sequencing of *L. acidophilus* NCFM has demonstrated the presence of *bshA* and *bshB* genes in it. The predicted genetic sequence of the BSH as encoded by these loci has been demonstrated to share high homology with BSH enzymes present in other *Lactobacillus* sp., indicating that these *bshA* and *bshB* have different substrate specificities, and might have been acquired from diverse origins [51]. Together, these reports indicate the presence of BSH in all the *Bifidobacterium* and *Lactobacillus* strains particularly those of GI origin; however, it is plausible that these strains might also transfer the *bsh* genes to other gut habitants such as *Listeria monocytogenes*.

## 3. Functions of bsh

Although the exact functions of bacterial BSH enzymes remain uncomprehended, researchers investigating diverse aspects of microbial BSHs particularly in context to specific nutritional and physiological activities have proposed several hypothesizes and speculations, as summarized herein.

### 3.1. Nutritional Role

The ability of the probiotics to degrade bile salts is one of the important criteria for their selection as dietary or food supplements. As reviewed by Begley et al. [52], the amino acids released due to probiotic-mediated bile salt deconjugation can be utilized as a source of carbon, nitrogen, and/or energy by other gut microbes. Therefore, deconjugation of bile salts by probiotics can offer nutritional opportunities to other microbes colonizing the gut lumen. It has been demonstrated that certain BSH-utilizing *Clostridium* sp. could utilize taurine as an electron receptor. In addition, the taurine as well as taurine-conjugated bile salts have also been associated with the improved growth of *Clostridium* strains [53, 54]. Moreover, the transcription of *bsh* genes of genus *Bifidobacterium longum* has been attributed to a *glnE* gene homolog encoding glutamine synthetase adenylyltransferase (GlnE), an enzyme component of the nitrogen regulation process [55]. However, in contrast, some *Lactobacillus* strains have been found to be unable to utilize bile salts as cellular precursor [56, 57].

### 3.2. Alteration of Membrane Characteristics

Intestinal innate immunity is presented by the antibacterial enzymes (e.g., lysozyme, phospholipase A2, and *α*-defensins) that antagonize unsolicited invading microorganisms. However, the extent of damage by these host defenses depends on several factors including the composition and chemical nature, hydrophobicity, permeability, and fluidity of the bacterial cell membrane. One of the posited functions of BSHs is facilitating the coprecipitation of cholesterol along with unconjugated bile acids, or incorporation of cholesterol or bile moieties into the bacterial cell membranes [58–60], thereby plausibly enhancing the malleable strength of the bacterial cell membranes [61] or amending the cellular fluidity. In addition, BSH-mediated changes in the bacterial cell surface may potentially stimulate safeguard against disruptions in the structure/integrity of bacterial cell membranes induced by the host immunity or immune system. The resistance mechanisms may be helpful for pathogenic bacteria to establish persistent infections. Therefore, commensal microbes possessing BSH enzymes may be selected and utilized to mitigate against specific pathogenic bacteria.

### 3.3. Bile Detoxification

There is a well-established link between bile salt hydrolysis bile salt and tolerance in multiple research studies carried out using *bsh* mutant and wild-type probiotic strains. For example, a *L.* mutant *amylovorus* isolated by *N*-methyl-*N*^1^-nitro-*N*-nitrosoguanidine mutagenesis and possessing partially reduced BSH activity exhibits reduction in growth in culture media supplemented with the bile salts [62]. Also, *L. plantarum* [8] and *Listeria monocytogenes* [40, 41] mutants for *bsh* genes were sensitive to bile salts. Though the information is scarce on exact mechanism of BSH in conferring bile tolerance, it has been speculated that the protonated (nondissociated) kinds of bile salts may demonstrate toxicity by intracellular acidification, in a way analogous to organic acids. The cells possessing BSH activity could protect themselves by forming or developing weaker unconjugated bile salts [8]. This could be helpful in reversing the pH drip by recapturing followed by exportation of the cotransported protons. The glycoconjugated to tauroconjugated bile salt ratio in human bile is generally 3 : 1. Several *in vitro* studies have shown that while tauroconjugated bile salts typically have none to only slight effect on bacterial cells at different pH, glycoconjugated bile salts are highly toxic at an acidic range of pH, which may explain why *bsh* mutants are inhibited more strongly compared to the parent cell counterparts [8, 40, 41]. Therefore, BSHs have been suggested to be of particular significance in protection against the toxic effects of glycoconjugated bile salts especially at the low pH. In addition, the BSH activity is specifically important particularly at the port of (a) entry of bile into the duodenum and (b) occurrence of acid reflux from the stomach and/or (c) in specific localized intestinal compartments wherein LAB lowers the pH. This can be corroborated by the findings that the glycoconjugated bile salts are preferentially hydrolyzed by BSHs [63, 64] and that the pH optima for BSHs is slightly acidic (pH 5.0 to 6.0) [45, 65].

### 3.4. Persistence in the Gastrointestinal Tract

Given that the BSHs may confer protection against the harmful effects of bile and elements or modules of the innate immune system (e.g., defensins) through cell surface alterations, it is reasonable to conceive their possible role the survival and/or persistence of bacterial strains within the GI tract. In a study comparing the abilities of three *Lactobacillus* strains *in vitro*, Bateup et al. [66] reported a wide array of BSH activity in these strains (high activity in one strain and moderate activity in second strain, while no activity in the third one) to colonize the gut of a mouse model completely devoid of LAB in their GI tract. Intriguingly, enumerating the gut *Lactobacillus* population two weeks postinoculation showed comparable and efficient colonization by all the strains, thereby indicating that BSH may not be a requisite for the intestinal colonization of these and similar bacterial strains. Another study, however, convincingly evidenced the contribution of BSH in the persistence of *Listeria monocytogenes*, wherein a *bsh* mutant when orally infected in guinea pigs was found to show 4-5 log decline in the fecal carriage of bacterial count of the mutant strains versus the control/parent strain after 48 h of experimental treatment. In addition, inserting an extra copy of the gene (on a plasmid) in to the cells could increase the multiplication of the parent strain in the intestinal tract by up to 10-fold, thereby demonstrating the importance of BSH in bacterial persistence in the GI tract [67].

Notably, the disparity in the conclusions of these two studies may be attributed to the two main differences between these two studies [66, 67]. Firstly, the two studies compared the isogenic *L. monocytogenes* wild-type and *bsh* mutant strains, and hence, it is likely that the contribution of BSH in bacterial strain survival in the gut was masked by the intrinsic strain-specific differences in lactobacilli used in the other study. Secondly, the study by Bateup et al. [66] employed LAB-devoid mouse model and showed that the role for BSH might be revealed in a more competitive intestinal environment. Therefore, further studies are needed to focus on determining the universal function of BSH with particular reference to its role in the persistence of clinically important probiotic strains (e.g., bifidobacteria and lactobacilli) in the GI tract.

### 3.5. Impact of Microbial BSH Activity on the Host

#### 3.5.1. Cholesterol Reduction

Hypercholesterolemia is one of the major risk factors for CHD development. Several pharmacologic formulations or drugs such as statins and bile acid sequestrants are widely available, but these often present unwanted side effects and suboptimal efficacy in humans [68]. Several orally administered probiotic strains have been demonstrated to be able to confer as much as 22-33% reduction in the cholesterol levels [7, 23] and even prevent hypercholesterolemia in high-fat diet-fed murine models [69]. These hypocholesterolemic effects could be attributed at least partly to the BSH activity, although other beneficial attributes such as cholesterol assimilation by specific gut microbes, cholesterol binding on to the bacterial cell wall, and/or the various beneficial physiological effects of microbial metabolites such as short-chain fatty acids (SCFA) might also underlie these effects (Figure 2) [65]. The reabsorption of deconjugated bile salts is less efficient compared to that of conjugated bile salts, due to which the free bile acids are excreted in larger quantities in the feces. In addition, the intestinal solubilization and absorption of lipids are also less efficient for free bile salts. Consequently, the deconjugation of bile salts might cause reduction in serum cholesterol levels (a) by raising the demand for cholesterol required for the *de novo* synthesis of bile acids to compensate for those lost via feces or (b) by lowering the cholesterol solubility and subsequent absorption through the gut lumen.

*(1) Impaired Digestive Functions*. Compared to conjugated bile acid molecules, the unconjugated bile acids are less efficacious in emulsifying dietary lipids and micelle formation; hence, the normal process of lipid digestion may be compromised by the BSH activity thereby also leading to the impaired absorption of fatty acids and monoglycerides [29]. Microbial BSH activity is related to growth defects noticed in experimental poultry birds [70], but not in mice [66].

### 3.6. Impact of Probiotics Supplementation on Plasma Lipid Profile

The concept of the prohealth benefits of fermented foods in humans goes back to as early as the 19^th^ century, when Elie Metchnikov postulated that microbially fermented milk “prevented intestinal putrefaction” and “helped maintain the forces of the body” (cited in [22, 71]). The persons consuming diets rich in fiber and beneficial phytometabolites have gut microbiota with powerful metabolic activities. The impact of commonly used probiotics and major findings on cholesterol levels are summarized in Table 2. A study conducted on Maasai tribal subjects with low serum lipid profile revealed that despite consuming ample meat, the Maasai people scarcely suffer from CHD. They consume 4-5 liters of fermented whole milk on a daily basis. This urged the scientists to investigate the impact of fermented milk on cholesterol levels in the blood [72]. Another study involving 26 volunteers inferred that consumption of high amounts of LAB-containing yoghurt had adverse effects on cholesterolemia [73]. It is likely that some strains of *L. acidophilus* help binding of cholesterol to intestinal lumen cells, thus decreasing its absorption into circulatory system [31]. Tahri et al. [25], while studying the cholesterol assimilation by *Bifidobacterium* strains, noted that elimination of cholesterol from microbial culture medium could be due to growing bacteria.

Lin and Chen [74] studies hypocholesterolemic ability of probiotic grade six *L. acidophilus* strains *in vitro* and inferred that cholesterol-lowering activity of *L. acidophilus* ATCC4356 was due to cholesterol assimilation and adsorption to bacterial cells. Grunewald [75] noted a decline in serum cholesterol levels in experimental rats fed *L. acidophilus* fermented. It was inferred that consumption of probiotics or probiotic-fermented food might assist in reducing the serum cholesterol. The factors responsible for hypocholesterolemic activity were released during fermentation of the milk.

Feeding of low doses (10^4^ cells/day) of probiotic *L. reuteri* CRL 1098 could induce about 38% reduction in blood triglycerides, 40% reduction in cholesterol, and 20% increase in the ratio of HDL : LDL in hypercholesterolemic murine models. The data imply that *L. reuteri* CRL 1098 could serve as a hypocholesterolemic adjuvant [76]. Xiao et al. [77] concluded that drinking fermented milk containing probiotic *B. longum* BL1 caused a significant decrease in serum total cholesterol, triglycerides and LDL. Abd El-Gawad et al. [78] noted an inverse relationship between fecal excretion of bile acids and levels of serum cholesterol from a study conducted on rats fed cholesterol-enriched diets and buffalo milk yoghurt and soy-yoghurt having *Bifidobacterium lactis* Bb-12 or *B. longum* Bb-46. Kumar et al. [17] also explored hypocholesterolemic effect of *L. plantarum* in Sprague-Dawley rat models. In another study, 17 patients with type II hyperlipidemia were given *Lactobacillus sporogenes* for three months in an open-level fixed dose trial. A significant decrease in their serum cholesterol, LDL, and total cholesterol was noted, while HDL cholesterol increased marginally [79].

Anderson and Gilliland [80] examined the effect of yoghurt having *L. acidophilus* L1 on serum lipids during a controlled clinical trial in hypercholesterolemic humans and observed a 2.4% decrease in serum cholesterol in persons. It was concluded that *L. acidophilus* L1 fermented milk can reduce the risk of CHD by 6-10%. A placebo-controlled, double-blinded, randomized crossover study demonstrated that a blend of *L. acidophilus* 74-2 and *B. animalis* subsp. lactis DGCC 420 augmented immune response and could concomitantly reduce serum cholesterol levels by 11.6% during the experimental trial [81].

Genetically modified *L. plantarum* 80 (pCBH1) could degrade and remove conjugated bile acids though BSH action and paved the way to lower the cholesterol levels using genetically modified probiotics [82].

### 3.7. Plasma Lipoprotein Synthesis and Metabolism

The intestine and liver are primarily responsible for the synthesis and transport of lipoproteins. The liver synthesizes the bile which is moved to and stored in the gallbladder. The bile salts get into action and poured into the small intestine in response to fats in meal, thereby making their digestion and absorption practicable. Fatty acids, triglycerides, and cholesterol combine in the gut epithelial cells and collectively constitute the chylomicrons [83]. The chylomicrons enter the blood through the lymphatic system, find their way to the liver, and are converted triglycerides and cholesterols. Bile salts enter the ileum; majority of them are again absorbed and enter the blood and reach the liver again. The bile salts remain in bile for the next rounds of their use. Unabsorbed bile salts are finally eliminated as a part of defecation. Hepatic cells synthesize cholesterol and therefore contribute to cholesterol reservoir of the body.

#### 3.7.1. Biosynthesis of Cholesterol

It is evident that approximately 50% of the total cholesterol is produced inside the body, of which10% is synthesized in the liver and 15% is in generated in the intestine [83]. Microsomes and cytoplasm are the prime sites of cholesterol biosynthesis via the following steps [84, 85]:

- Conversion of Acetyl-CoAs to HMG-CoA, i.e., 3-hydroxy-3-methylglutaryl-CoA [85]
- Transformation of HMG-CoA to mevalonate
- Conversion of mevalonate to isopentenyl pyrophosphate
- Conversion of isopentenyl pyrophosphate to squalene
- Change of squalene to cholesterol

#### 3.7.2. Regulation of Cholesterol Biosynthesis

While around 1 gram of cholesterol is produced in healthy adult humans, 0.3 gram of it is utilized daily. A moderately persistent level of cholesterol (150-200 mg/dL) is maintained in the human body by managing its levels of *de novo* synthesis [86]. The dietary as well as synthesized cholesterol participates in membrane formation as well as synthesis of bile acids and steroid hormones required on day-to-day basis [87]. According to Kaplan and Pesse [83] and Omoigui [86], the following three mechanisms regulate the cholesterol supply:

- Regulation of HMG-CoA reductase (HMGR)
- Regulation of extra intracellular free cholesterol via Acyl-CoA cholesterol acyltransferase (ACAT) [86]
- Regulation plasma cholesterol through HDL-mediated reverse transport, and LDL receptor-mediated uptake

Hepatic cholesterol reservoir is important for two reasons: (i) the liver utilizes part of cholesterol to synthesize bile salts which are transported to the gallbladder and partly poured into the gut. The bile salts poured into gut help the emulsification of dietary fats, followed by their hydrolysis and absorption; (ii) the remaining cholesterol is utilized for other functions in the body system. For this, the liver combines cholesterol from its pool with triglycerides and covers it with a particular protein so that it is dissolved and enters the blood.

The liver then drains the associated somewhat large molecules, known as very low-density lipoproteins (VLDL) into the blood circulatory system. Lipoprotein lipase (LPL) found in the walls of the arteries assists in the removal of triglycerides from VLDL. During the process, the VLDLs shrivel in size; hence, intermediate density lipoproteins (IDL) represent the relatively larger portion of VLDLs.

#### 3.7.3. Low-Density Lipoprotein (LDL)

As a continued process of separation, low-density lipoprotein (LDL) is left behind. The lipoprotein still maintains a hefty extent of cholesterol. The protein layer assists the body cell transport LDL through LDL receptors. Cholesterol is taken away from LDL in some tissues like the liver and inner layer of arteries. Resulting free radicals are highly reactive and oxidative in nature which oxidize LDL cholesterol and cause the development of atherosclerotic arterial plaques. Antioxidants, whether taken orally or formed from dietary ingredients, are crucial to prevent oxidative stress and atherosclerosis [88].

#### 3.7.4. High-Density Lipoprotein (HDL)

In addition to above metabolites, the liver synthesizes another form of lipoprotein, called HDL. The HDL has little triglyceride and cholesterol and has a characteristic protein covering. HDL collects the leftover cholesterol remaining unutilized. An enzyme, named lecithin-cholesterol acyl transferase (LCAT), assists in transportation of the unutilized cholesterol back to the HDL. In addition, HDL absorbs the unused cholesterol from the arteries, liver, and other tissues. LCAT and HDL cholesterol serve to scavenge a fraction of oxidized LDL. HDL circulates in the body and collects cholesterol from various tissues, returns back to the liver, and accumulates the liver cholesterol pool.

*(1) Apo A-I*. Apo-A-1 is the key apolipoprotein in HDL cholesterol. It has antioxidant and anti-inflammatory activities and assists in the collection of extra cholesterol from outer cells and transporting it back it to the liver [89].

The Apo33 B/Apo-A ratio is an important clinical indicator of cardiovascular risk. According to Walldius et al. [90], the higher the ratio, the higher is the possibility of cholesterol amassing in arteries.

*(2) Apo B*. The Apo B is found in atherogenic particles: VLDL, IDL, and LDL cholesterol. All of them have at least one Apo-B inside them; hence, the number or quantity of Apo B is an indirect indicator of the number of the above atherogenic particles. Apo B assists in capturing these particles from the arterial lumen and walls. On the other hand, Apo B generated in the liver helps in the stabilization and relocation of cholesterol and triglycerides in plasma IDL, VLDL, and sd-LDL. Of all Apo B particles existing in the blood, more than 90% do exist in the form of low-density lipid cholesterol. Hence, the Apo-B/Apo-A ratio is utilized as an indicator of cardiovascular risk. The higher the ratio, the higher the probability of cholesterol deposits in the arteries [90].

### 3.8. Probiotics Actions on Lipids

Certain anaerobes in the gut ferment dietary indigestible saccharides or fiber and enhance level of SCFAs [71, 91]. The fermentation is catalyzed by a cartel of hydrolytic microbial enzymes. Notably, formation of SCFAs is a good indication of health gut ecosystem.

Approximately 100 to 450 mmol of the short chain fatty acids (SCFAs) is produced in the large intestine daily with relative proportions of acetate, propionate, and butyrate being approximately 60 : 20 : 15. The production depends on the kind of dietary ingredients and the gut microbial consortia [23, 71]. Acetate boosts total cholesterol while propionate increases glucose levels in the blood and assists in reducing hypercholesterolemia induced by the acetate. Propionate does so by reducing the use of acetate for cholesterol and fatty acid synthesis. In addition, SCFAs serve as the potential modulators of food intake and energy sensing process into the brain that might have an indirect role in reducing cholesterol and other metabolisms [92].

The probiotics prevent the formation of micelles, which assist in the absorption of cholesterol from the intestine. Cholesterol is also dealt in the same manner when it enters in the enterohepatic circulation. Various strains of probiotic LAB breakdown bile acids and hydrolyze bile salts by means of hydroxy steroid dehydrogenase and BSH. This interrupts the enterohepatic circulation of bile acids [93–95].

Hydroxy methyl glutarate CoA (HMG CoA), an intermediate formed from acetyl CoA and acetoacetyl CoA catalyzed by HMG-CoA synthase, helps the probiotics to obstruct HMG-CoA reductase catalytic function. This serves as a rate-limiting biocatalyst in endogenous synthesis of cholesterol. Probiotics control the absorption of cholesterol from the gut lumen, hence minimizing the levels of free cholesterol in serum. Cholesterol is also assimilated during the progression of *L. acidophilus*, as permeability of the cells to biles as well *β*-galactosidase activity of *L. acidophilus* was found to increase in the presence of ox gall [96].

### 3.9. Mechanisms of Cholesterol-Reduction and Their Consequences

Reducing cholesterol is one of the most desirable features of probiotic strains intended for human applications. As cited above, deconjugation of bile acids catalyzed by probiotics BSH is one of the key steps of lowering the cholesterol. Bile, the resulting metabolites of cholesterol in the liver, is released into the duodenum where it is mixed with semidigested food [52]. Once deconjugated, the bile acids being the least soluble are eventually eliminated with feces. While evaluating the role of BSH of a cholesterol-lowering probiotic strain, *L. plantarum* 80 (pCBH1), it was inferred that BSH activity is imperative to hydrolyze conjugated forms of bile acids [82, 97].

Hypocholesterolemic properties of probiotics are also partly due to the capacity of specific probiotic candidates to remove cholesterol present in the intestinal lumen. For instance, *L. gasseri* has been found to remove cholesterol from culture media via binding of cholesterol to bacterial cells surfaces [27]. However, the exclusion of cholesterol by binding to cells varies among different strains and appears to be highly strain-specific [98]. Interestingly, in addition to live probiotic bacteria and the nongrowing bacteria, i.e., live probiotic bacteria suspended in phosphate buffer, the heat-killed cells have also been found to remove the cholesterol from culture media demonstrating that the cell surface might also contribute to its exclusion. Further, several strains of lactococci could also demonstrate the removal of cholesterol from the microbial culture media [98].

Alteration in fatty acid profile is observed for several probiotic bacteria growing *in vitro* in medium containing or devoid of supplemented cholesterol. The abundance of lipids in the cell membrane of probiotic strains suggests that the cholesterol is incorporated into bacterial cell membrane and may alter the fatty acid profile and membrane strength against lysis of bacteria [99, 100]. Using fluorescence probes analysis, it has been revealed that cholesterol accumulates more in phospholipid tails and the polar heads of the membrane phospholipid bilayer in probiotic bacteria growing in the presence of cholesterol.

Another route of excluding the cholesterol from the body system is the transformation of cholesterol into coprostanol and then the excretion of coprostanol though feces. This reduces the amount of cholesterol available for absorption into the body, thereby ultimately leading to reduced cholesterol levels. To evaluate the transformation of cholesterol into coprostanol in the presence of probiotic bacteria, Chiang et al. [101] have shown that cholesterol dehydrogenase/isomerase produced by bacteria such as *Sterolibacterium denitrificans* catalyzes the transformation of cholesterol to cholest-4-en-3-one during biotransformation of cholesterol to coprostanol. Lye et al. [100] examined the transformation of cholesterol to coprostanol by LAB wherein bacterial strains examined were found to possess intracellular as well as extracellular cholesterol reductase, thereby verifying the intracellular as well as extracellular transformation of cholesterol to coprostanol. Amount of the total cholesterol was found to decrease, and levels of coprostenol increased in the culture medium used to grow the LAB.

Nevertheless, this topic still requires further verifications particularly because administering the cholesterol reductase (intended to lower the serum cholesterol) also changes the cholesterol to coprostanol in the intestine. Additionally, with few exceptions, most of the conclusions and hypotheses are drawn mainly on the basis of *in vitro* studies. Most studies are focused primarily on unraveling merely the plausible hypocholesterolemic mechanisms merely. Using hypercholesterolemic pigs as a model, Liong et al. [102] examined the hypocholesterolemic ability of a synbiotic formulation and the mechanisms underlying the hypocholesterolemic effects. It was observed that orally administered *L. acidophilus* ATCC 4962, fructo-oligosaccharides, mannitol, and inulin reduced the plasma total cholesterol, LDL cholesterol, and triacylglycerols in treated animals compared to nonadministered (control) animals. Further subfractioning and characterization of lipoproteins revealed that the synbiotics-treated pigs had lower amounts of cholesteryl esters in LDL particles, along with higher amounts of triacylglycerol. The triacylglycerol-enriched LDL particles were more vulnerable to enzymatic hydrolysis and exclusion from blood circulation. In addition, it was reported that the synbiotic supplementation could enhance cholesteryl esters in HDL fraction.

Remarkably, the HDL in view of its role in carrying cholesterol to hepatic cells during further hydrolysis is regarded as beneficial cholesterol. Therefore, it was also indicated that the synbiotics-induced hypocholesterolemic effects might be conferred via modulation of the cascade of cholesteryl esters and lipoprotein transporters. The prebiotics, such as inulin and fructo-oligosaccharides, being soluble, but indigestible can exhibit hypocholesterolemia via two mechanisms: by impairing cholesterol absorption plus removal through feces and/or increased synthesis or generation of SCFAs during enteric fermentation of dietary fiber (Figure 2) [103]. Orally administered inulin for 4 weeks decreased the serum LDL cholesterol and increased serum HDL cholesterol in hypercholesterolemic rats [104]. In comparison to the control groups, the rats receiving inulin had significantly more excretion of fecal lipids and cholesterol.

Analogous to indigestible fibers, soluble indigestible prebiotics enhance the viscosity of the contents in digestive tract and augment the thickness of the unstirred small intestine layer, thereby thwarting or preventing the uptake of cholesterol [105]. This might also lead to enhanced hepatic cholesterol catabolism could ultimately add to the hypocholesterolemic effects.

### 3.10. Future Prospects and Conclusions

Probiotics have recently received considerable attention in view of their proclaimed health benefits such as the improvement of lactose tolerance, resistance to infectious diseases, and suppression of inflammatory diseases and cancer. Reduction in serum cholesterol levels is one of the enviable benefits of probiotics. Considerable interest is noted in the use and effectiveness of probiotics to lower the serum lipids. Despite these claimed benefits from human clinical studies, a decisive and evidence-based outcome has not been achieved. Also, the precise mechanism(s) underlying the cholesterol-lowering or cholesterol-removal effects are still not clearly and wholly understood. Several possible mechanisms, such as cholesterol assimilation by growing-bacteria, cholesterol binding on to the probiotic cells, cholesterol integration into the host cell membrane, BSH-mediated bile deconjugation, and cholesterol coprecipitation with deconjugated bile, have been proposed. However, most of these mechanisms are highly strain-specific (and maybe host-specific as well) and the conditions generated in the laboratory may not always be practical or translatable in *in vivo* systems. The discrepancies in the data of different effects on serum cholesterol levels may be due differences in genus, species, and LAB strains used in different studies. Though hypocholesterolemic mechanisms of probiotics are not yet completely understood, the close association between cholesterol and bile salt metabolism is well established. The “BSH hypothesis” is put forward to explain the cholesterol-lowering effects of probiotics. The hypocholesterolemic attributes of specific probiotics exhibiting high BSH activities *in vitro* have been validated in some human and model animal studies. Moreover, considering that many commercial probiotic strains possess high BSH activities, further studies are indispensable to validate whether this BSH function of the probiotic strains is beneficial or harmful for the host. In probiotic science and research milieus, bile tolerance is of utmost significance while selecting the probiotic strains because tolerance to bile enhances the adaptation or capacity of probiotics during their transit along the duodenum and eventually to colonize the gut epithelium. Thus, it is important to understand the physiological and molecular mechanisms by which enteric microorganisms including lactobacilli and bifidobacteria have evolved to resist against antimicrobial activity of bile in the GI tract. A further study on conserved as well as variable regions of the *bsh* genes from bacterial species and strains with probiotic importance will facilitate the development of phylogenetic markers other bacteria. Furthermore, one of the future challenges would also be to understand the physiological impacts of BSH activity on the bacteria producing this enzyme as well as on the host cells.

**Conflicts of Interest**

The authors declare that they have no conflicts of interest.

**References**

[1] M. Kumar, R. Nagpal, R. Kumar et al., “Cholesterol-Lowering Probiotics as Potential Biotherapeutics for Metabolic Diseases,” *Experimental Diabetes Research*, vol. 2012, Article ID 902917, 14 pages, 2012.

[2] WHO, *Cardiovascular Disease; Fact Sheet N°317*, Geneva, Switzerland, 2009, May 2010, http://www.who.int/mediacentre/factsheets/fs317/en/print.html.

[3] S. Yusuf, S. Hawken, S. Ounpuu et al., “Effect of potentially modifiable risk factors associated with myocardial infarction in 52 countries (the INTERHEART study): case-control study,” *Lancet*, vol. 364, no. 9438, pp. 937–952, 2004.

[4] WHO, *Diet, Nutrition and Prevention of Chronic Diseases*, Report of a Joint WHO/FAO Expert Consultation, Geneva, Switzerland, 2003.

[5] S. Dunn-Emke, G. Weidner, and D. Ornish, “Benefits of a low-fat plant-based diet,” *Obesity Research*, vol. 9, no. 11, p. 731, 2001.

[6] E. G. Bliznakov, “Lipid-lowering drugs (statins), cholesterol, and coenzyme Q_10_. The Baycol case - a modern Pandora's box,” *Biomedicine & Pharmacotherapy*, vol. 56, no. 1, pp. 56–59, 2002.

[7] I. De Smet, P. De Boever, and W. Verstraete, “Cholesterol lowering in pigs through enhanced bacterial bile salt hydrolase activity,” *The British Journal of Nutrition*, vol. 79, no. 2, pp. 185–194, 1998.

[8] I. De Smet, L. Van Hoorde, M. Vande Woestyne, H. Christiaens, and W. Verstraete, “Significance of bile salt hydrolytic activities of lactobacilli,” *The Journal of Applied Bacteriology*, vol. 79, no. 3, pp. 292–301, 1995.

[9] T. A. B. Sanders, “Food production and food safety,” *BMJ*, vol. 318, no. 7199, pp. 1689–1693, 1999.

[10] M. Kumar, P. V. Behare, D. Mohania, S. Arora, A. Kaur, and R. Nagpal, *Health-Promoting Probiotic Functional Foods: Potential and Prospects*, vol. 20, Agrofood Industry Hi-Tech, 2009.

[11] R. Nagpal, H. Yadav, A. K. Puniya, K. Singh, S. Jain, and F. Marotta, “Potential of probiotics and prebiotics for synbiotic functional dairy foods,” *International Journal of Probiotics and Prebiotics*, vol. 2, pp. 75–84, 2007.

[12] S. Bengmark, “Immunonutrition: role of biosurfactants, fiber, and probiotic bacteria,” *Nutrition*, vol. 14, no. 7-8, pp. 585–594, 1998.

[13] M. Kumar, A. Kumar, R. Nagpal et al., “Cancer-preventing attributes of probiotics: an update,” *International Journal of Food Sciences and Nutrition*, vol. 61, no. 5, pp. 473–496, 2010.

[14] M. Kumar, D. Mohania, D. Poddar et al., “A probiotic fermented milk prepared by mixed culture combination reduces pathogen shedding and alleviates disease signs in rats challenged with pathogens,” *International Journal of Probiotics and Prebiotics*, vol. 4, no. 3, pp. 211–218, 2009.

[15] M. Kumar, V. Verma, R. Nagpal et al., “Anticarcinogenic effect of probiotic fermented milk and chlorophyllin on aflatoxin-B1-induced liver carcinogenesis in rats,” *The British Journal of Nutrition*, vol. 107, no. 7, pp. 1006–1016, 2012.

[16] M. Kumar, V. Verma, R. Nagpal et al., “Effect of probiotic fermented milk and chlorophyllin on gene expressions and genotoxicity during AFB_1_-induced hepatocellular carcinoma,” *Gene*, vol. 490, no. 1-2, pp. 54–59, 2011.

[17] R. Kumar, S. Grover, and V. K. Batish, “Hypocholesterolaemic effect of dietary inclusion of two putative probiotic bile salt hydrolase-producing Lactobacillus plantarum strains in Sprague-Dawley rats,” *The British Journal of Nutrition*, vol. 105, no. 4, pp. 561–573, 2011.

[18] D. R. Mack, S. Michail, S. Wei, L. McDougall, and M. A. Hollingsworth, “Probiotics inhibit enteropathogenic E. coliadherence in vitro by inducing intestinal mucin gene expression,” *The American Journal of Physiology*, vol. 276, no. 4, pp. G941–G950, 1999.

[19] H. Yadav, S. Jain, and P. R. Sinha, “Formation of oligosaccharides in skim milk fermented with mixed dahi cultures, Lactococcus lactis ssp diacetylactis and probiotic strains of lactobacilli,” *The Journal of Dairy Research*, vol. 74, no. 2, pp. 154–159, 2007.

[20] H. Yadav, S. Jain, and P. R. Sinha, “Antidiabetic effect of probiotic dahi containing *Lactobacillus acidophilus* and *Lactobacillus casei* in high fructose fed rats,” *Nutrition*, vol. 23, no. 1, pp. 62–68, 2007.

[21] K. Niedzielin, H. Kordecki, and B. Birkenfeld, “A controlled, double-blind, randomized study on the efficacy of Lactobacillus plantarum 299V in patients with irritable bowel syndrome,” *European Journal of Gastroenterology & Hepatology*, vol. 13, no. 10, pp. 1143–1147, 2001.

[22] R. Nagpal, P. V. Behare, M. Kumar et al., “Milk, milk products, and disease free health: an updated overview,” *Critical Reviews in Food Science and Nutrition*, vol. 52, no. 4, pp. 321–333, 2012.

[23] D. I. A. Pereira and G. R. Gibson, “Effects of consumption of probiotics and prebiotics on serum lipid levels in humans,” *Critical Reviews in Biochemistry and Molecular Biology*, vol. 37, no. 4, pp. 259–281, 2008.

[24] F. A. Klaver and R. van der Meer, “The assumed assimilation of cholesterol by Lactobacilli and Bifidobacterium bifidum is due to their bile salt-deconjugating activity,” *Applied and Environmental Microbiology*, vol. 59, no. 4, pp. 1120–1124, 1993.

[25] K. Tahri, J. P. Grill, and F. Schneider, “Bifidobacteria strain behavior toward cholesterol: coprecipitation with bile salts and assimilation,” *Current Microbiology*, vol. 33, no. 3, pp. 187–193, 1996.

[26] K. Tahri, J. P. Grill, and F. Schneider, “Involvement of trihydroxyconjugated bile salts in cholesterol assimilation by bifidobacteria,” *Current Microbiology*, vol. 34, no. 2, pp. 79–84, 1997.

[27] A. H. Usman, “Bile tolerance, taurocholate deconjugation, and binding of cholesterol by Lactobacillus gasseri strains,” *Journal of Dairy Science*, vol. 82, no. 2, pp. 243–248, 1999.

[28] B. Z. De Rodas, S. E. Gilliland, and C. V. Maxwell, “Hypocholesterolemic action of *Lactobacillus acidophilus* ATCC 43121 and calcium in swine with hypercholesterolemia induced by diet,” *Journal of Dairy Science*, vol. 79, no. 12, pp. 2121–2128, 1996.

[29] I. De Smet, L. Van Hoorde, N. De Saeyer, M. Vande Woestyne, and W. Verstraete, “*In vitro* study of bile salt hydrolase (BSH) activity of BSH isogenic *Lactobacillus plantarum* 80 strains and estimation of cholesterol lowering through enhanced BSH activity,” *Microbial Ecology in Health & Disease*, vol. 7, pp. 315–329, 1994.

[30] M. O. Reynier, J. C. Montet, A. Gerolami et al., “Comparative effects of cholic, chenodeoxycholic, and ursodeoxycholic acids on micellar solubilization and intestinal absorption of cholesterol,” *Journal of Lipid Research*, vol. 22, no. 3, pp. 467–473, 1981.

[31] S. E. Gilliland, C. R. Nelson, and C. Maxwell, “Assimilation of cholesterol by Lactobacillus acidophilus,” *Applied and Environmental Microbiology*, vol. 49, no. 2, pp. 377–381, 1985.

[32] M. du Toit, C. M. Franz, L. M. Dicks et al., “Characterisation and selection of probiotic lactobacilli for a preliminary minipig feeding trial and their effect on serum cholesterol levels, faeces pH and faeces moisture content,” *International Journal of Food Microbiology*, vol. 40, no. 1-2, pp. 93–104, 1998.

[33] D. K. Walker and S. E. Gilliland, “Relationships among bile tolerance, bile salt deconjugation, and assimilation of cholesterol by *Lactobacillus acidophilus*,” *Journal of Dairy Science*, vol. 76, no. 4, pp. 956–961, 1993.

[34] M. Rojas, F. Ascencio, and P. L. Conway, “Purification and characterization of a surface protein from Lactobacillus fermentum 104R that binds to porcine small intestinal mucus and gastric mucin,” *Applied and Environmental Microbiology*, vol. 68, no. 5, pp. 2330–2336, 2002.

[35] C. Gusils, S. N. González, and G. Oliver, “Some probiotic properties of chicken lactobacilli,” *Canadian Journal of Microbiology*, vol. 45, no. 12, pp. 981–987, 1999.

[36] C. Heinemann, J. E. van Hylckama Vlieg, D. B. Janssen, H. J. Busscher, H. C. van der Mei, and G. Reid, “Purification and characterization of a surface-binding protein from Lactobacillus fermentum RC-14 that inhibits adhesion of Enterococcus faecalis 1131,” *FEMS Microbiology Letters*, vol. 190, no. 1, pp. 177–180, 2000.

[37] G. Reid, A. W. Bruce, N. Fraser, C. Heinemann, J. Owen, and B. Henning, “Oral probiotics can resolve urogenital infections,” *FEMS Immunology and Medical Microbiology*, vol. 30, no. 1, pp. 49–52, 2001.

[38] M. C. Carey and W. C. Duane, “Enterohepatic circulation,” in *The Liver: Biology and Pathobiology*, I. M. Arias, N. Boyer, N. Fausto, W. B. Jackoby, D. A. Schachter, and D. A. Shafritz, Eds., pp. 719–738, Raven Press, Ltd., New York, NY, USA, 1994.

[39] A. F. Hofmann, “Bile acids,” in *The Liver: Biology and Pathobiology*, I. M. Arias, J. L. Boyer, N. Fausto, W. B. Jackoby, D. A. Schachter, and D. A. Shafritz, Eds., pp. 677–718, Raven Press, Ltd., New York, NY, USA, 1994.

[40] M. Begley, C. G. M. Gahan, and C. Hill, “The interaction between bacteria and bile,” *FEMS Microbiology Reviews*, vol. 29, no. 4, pp. 625–651, 2005.

[41] M. Begley, R. D. Sleator, C. G. M. Gahan, and C. Hill, “Contribution of three bile-associated loci, bsh, pva, and btlB, to gastrointestinal persistence and bile tolerance of Listeria monocytogenes,” *Infection and Immunity*, vol. 73, no. 2, pp. 894–904, 2005.

[42] Z. R. Vlahcevic, D. M. Heuman, and P. B. Hylemon, “Physiology and pathophysiology of enterohepatic circulation of bile acids,” in *Hepatology: A Textbook of Liver Disease*, Vol. 1, D. Zakim and T. Boyer, Eds., pp. 376–417, Saunders, Philadelphia, PA, USA, 3rd edition, 1996.

[43] O. Bortolini, A. Medici, and S. Poli, “Biotransformations on steroid nucleus of bile acids,” *Steroids*, vol. 62, no. 8-9, pp. 564–577, 1997.

[44] A. K. Batta, G. Salen, R. Arora, S. Shefer, M. Batta, and A. Person, “Side chain conjugation prevents bacterial 7-dehydroxylation of bile acids,” *The Journal of Biological Chemistry*, vol. 265, no. 19, pp. 10925–10928, 1990.

[45] G. Corzo and S. E. Gilliland, “Bile salt hydrolase activity of three strains of *Lactobacillus acidophilus*,” *Journal of Dairy Science*, vol. 82, no. 3, pp. 472–480, 1999.

[46] G. Corzo and S. E. Gilliland, “Measurement of bile salt hydrolase activity from *Lactobacillus acidophilus* based on disappearance of conjugated bile salts,” *Journal of Dairy Science*, vol. 82, no. 3, pp. 466–471, 1999.

[47] C. A. Elkins, S. A. Moser, and D. C. Savage, “Genes encoding bile salt hydrolases and conjugated bile salt transporters in Lactobacillus johnsonii 100-100 and other Lactobacillus species,” *Microbiology*, vol. 147, no. 12, pp. 3403–3412, 2001.

[48] C. M. Franz, I. Specht, P. Haberer, and W. H. Holzapfel, “Bile salt hydrolase activity of enterococci isolated from food: screening and quantitative determination,” *Journal of Food Protection*, vol. 64, no. 5, pp. 725–729, 2001.

[49] H. Tanaka, K. Doesburg, T. Iwasaki, and I. Mierau, “Screening of lactic acid bacteria for bile salt hydrolase activity,” *Journal of Dairy Science*, vol. 82, no. 12, pp. 2530–2535, 1999.

[50] D. R. Edgell, M. Belfort, and D. A. Shub, “Barriers to intron promiscuity in bacteria,” *Journal of Bacteriology*, vol. 182, no. 19, pp. 5281–5289, 2000.

[51] O. McAuliffe, R. J. Cano, and T. R. Klaenhammer, “Genetic analysis of two bile salt hydrolase activities in Lactobacillus acidophilus NCFM,” *Applied and Environmental Microbiology*, vol. 71, no. 8, pp. 4925–4929, 2005.

[52] M. Begley, C. Hill, and C. G. M. Gahan, “Bile salt hydrolase activity in probiotics,” *Applied and Environmental Microbiology*, vol. 72, no. 3, pp. 1729–1738, 2006.

[53] S. M. Huijghebaert, J. A. Mertens, and H. J. Eyssen, “Isolation of a bile salt sulfatase-producing Clostridium strain from rat intestinal microflora,” *Applied and Environmental Microbiology*, vol. 43, no. 1, pp. 185–192, 1982.

[54] J. Van Eldere, P. Celis, G. De Pauw, E. Lesaffre, and H. Eyssen, “Tauroconjugation of cholic acid stimulates 7alpha-dehydroxylation by fecal bacteria,” *Applied and Environmental Microbiology*, vol. 62, no. 2, pp. 656–661, 1996.

[55] H. Tanaka, H. Hashiba, J. Kok, and I. Mierau, “Bile salt hydrolase of Bifidobacterium longum-biochemical and genetic characterization,” *Applied and Environmental Microbiology*, vol. 66, no. 6, pp. 2502–2512, 2000.

[56] S. E. Gilliland and M. L. Speck, “Deconjugation of bile acids by intestinal lactobacilli,” *Applied and Environmental Microbiology*, vol. 33, no. 1, pp. 15–18, 1977.

[57] G. W. Tannock, M. P. Dashkevicz, and S. D. Feighner, “Lactobacilli and bile salt hydrolase in the murine intestinal tract,” *Applied and Environmental Microbiology*, vol. 55, no. 7, pp. 1848–1851, 1989.

[58] P. C. Dambekodi and S. E. Gilliland, “Incorporation of cholesterol into the cellular membrane of Bifidobacterium longum,” *Journal of Dairy Science*, vol. 81, no. 7, pp. 1818–1824, 1998.

[59] M. P. Taranto, F. Sesma, A. Pesce de Ruiz Holgado, and G. F. de Valdez, “Bile salts hydrolase plays a key role on cholesterol removal by Lactobacillus reuteri,” *Biotechnology Letters*, vol. 19, no. 9, pp. 845–847, 1997

[60] M. P. Taranto, M. L. Fernandez Murga, G. Lorca, and G. F. de Valdez, “Bile salts and cholesterol induce changes in the lipid cell membrane of Lactobacillus reuteri,” *Journal of Applied Microbiology*, vol. 95, no. 1, pp. 86–91, 2003.

[61] J. M. Boggs, “Lipid intermolecular hydrogen bonding: influence on structural organization and membrane function,” *Biochimica et Biophysica Acta (BBA) - Reviews on Biomembranes*, vol. 906, no. 3, pp. 353–404, 1987.

[62] J. P. Grill, C. Cayuela, J. M. Antoine, and F. Schneider, “Isolation and characterization of a Lactobacillus amylovorus mutant depleted in conjugated bile salt hydrolase activity: relation between activity and bile salt resistance,” *Journal of Applied Microbiology*, vol. 89, no. 4, pp. 553–563, 2000.

[63] J. P. Coleman and L. L. Hudson, “Cloning and characterization of a conjugated bile acid hydrolase gene from Clostridium perfringens,” *Applied and Environmental Microbiology*, vol. 61, no. 7, pp. 2514–2520, 1995.

[64] G. B. Kim, C. M. Miyamoto, E. A. Meighen, and B. H. Lee, “Cloning and characterization of the bile salt hydrolase genes (bsh) from Bifidobacterium bifidum strains,” *Applied and Environmental Microbiology*, vol. 70, no. 9, pp. 5603–5612, 2004.

[65] M. T. Liong and N. P. Shah, “Bile salt deconjugation ability, bile salt hydrolase activity and cholesterol co-precipitation ability of lactobacilli strains,” *International Dairy Journal*, vol. 15, no. 4, pp. 391–398, 2005.

[66] J. M. Bateup, M. A. McConnell, H. F. Jenkinson, and G. W. Tannock, “Comparison of Lactobacillus strains with respect to bile salt hydrolase activity, colonization of the gastrointestinal tract, and growth rate of the murine host,” *Applied and Environmental Microbiology*, vol. 61, no. 3, pp. 1147–1149, 1995.

[67] O. Dussurget, D. Cabanes, P. Dehoux et al., “Listeria monocytogenes bile salt hydrolase is a PrfA-regulated virulence factor involved in the intestinal and hepatic phases of listeriosis,” *Molecular Microbiology*, vol. 45, no. 4, pp. 1095–1106, 2002.

[68] H. Schuster, “Improving lipid management–to titrate, combine or switch,” *International Journal of Clinical Practice*, vol. 58, no. 7, pp. 689–694, 2004.

[69] M. P. Taranto, M. Medici, G. Perdigon, A. P. Ruiz Holgado, and G. F. Valdez, “Effect of *Lactobacillus reuteri* on the prevention of hypercholesterolemia in mice,” *Journal of Dairy Science*, vol. 83, no. 3, pp. 401–403, 2000.

[70] S. D. Feighner and M. P. Dashkevicz, “Effect of dietary carbohydrates on bacterial cholyltaurine hydrolase in poultry intestinal homogenates,” *Applied and Environmental Microbiology*, vol. 54, no. 2, pp. 337–342, 1988.

[71] M.-P. St-Onge, E. R. Farnworth, and P. J. H. Jones, “Consumption of fermented and nonfermented dairy products: effects on cholesterol concentrations and metabolism,” *The American Journal of Clinical Nutrition*, vol. 71, no. 3, pp. 674–681, 2000.

[72] G. V. Mann, “Studies of a surfactant and cholesteremia in the Maasai,” *The American Journal of Clinical Nutrition*, vol. 27, no. 5, pp. 464–469, 1974.

[73] G. V. Mann, “A factor in yogurt which lowers cholesteremia in man,” *Atherosclerosis*, vol. 26, no. 3, pp. 335–340, 1977.

[74] M.-Y. Lin and T.-W. Chen, “Reduction of cholesterol by Lactobacillus acidophilus in culture broth,” *Journal of Food and Drug Analysis*, vol. 8, no. 2, pp. 97–102, 2000.

[75] K. K. Grunewald, “Serum cholesterol levels in rats fed skim milk fermented by *Lactobacillus acidophilus*,” *Journal of Food Science*, vol. 47, no. 6, pp. 2078-2079, 1982.

[76] M. P. Taranto, M. Medici, G. Perdigon, A. P. Ruiz Holgado, and G. F. Valdez, “Evidence for hypocholesterolemic effect of *Lactobacillus reuteri* in hypercholesterolemic mice,” *Journal of Dairy Science*, vol. 81, no. 9, pp. 2336–2340, 1998.

[77] J. Z. Xiao, S. Kondo, N. Takahashi et al., “Effects of milk products fermented by *Bifidobacterium longum* on blood lipids in rats and healthy adult male volunteers,” *Journal of Dairy Science*, vol. 86, no. 7, pp. 2452–2461, 2003.

[78] I. A. Abd El-Gawad, E. M. El-Sayed, S. A. Hafez, H. M. El-Zeini, and F. A. Saleh, “The hypocholesterolaemic effect of milk yoghurt and soy-yoghurt containing bifidobacteria in rats fed on a cholesterol-enriched diet,” *International Dairy Journal*, vol. 15, no. 1, pp. 37–44, 2005.

[79] J. C. Mohan, R. Arora, and M. Khalilullah, “Preliminary observations on effect of Lactobacillus sporogenes on serum lipid levels in hypercholesterolemic patients,” *The Indian Journal of Medical Research*, vol. 92, pp. 431-432, 1990.

[80] J. W. Anderson and S. E. Gilliland, “Effect of fermented milk (yogurt) containing Lactobacillus acidophilus L1 on serum cholesterol in hypercholesterolemic humans,” *Journal of the American College of Nutrition*, vol. 18, no. 1, pp. 43–50, 1999.

[81] A. Klein, U. Friedrich, H. Vogelsang, and G. Jahreis, “*Lactobacillus acidophilus* 74-2 and *Bifidobacterium animalis* subsplactis DGCC 420 modulate unspecific cellular immune response in healthy adults,” *European Journal of Clinical Nutrition*, vol. 62, no. 5, pp. 584–593, 2008.

[82] M. L. Jones, H. Chen, W. Ouyang, T. Metz, and S. Prakash, “Microencapsulated genetically engineered *Lactobacillus plantarum* 80 (pCBH1) for bile acid deconjugation and its implication in lowering cholesterol,” *Journal of Biomedicine & Biotechnology*, vol. 2004, no. 1, pp. 61–69, 2004.

[83] L. A. Kaplan and A. J. Pesse, *Clinical Chemistry, Theory, Analysis, and Correlation*, Mosby Company, St. Louis, 3 edition, 1996.

[84] S. Dessi and B. Batetta, “Cholesterol metabolism in human tumors,” in *Cell Growth and Cholesterol Esters*, L. Bioscience, Ed., pp. 34–47, Kluwer Academic/Plenum Publishers, New York, NY, USA, 2003.

[85] J. Thomas, T. P. Shentu, and D. K. Singh, “Cholesterol: biosysthesis, functional diversity, homeostasis and regulation by natural products,” in *Biochemistry*, D. Eckinci, Ed., pp. 426–443, INTECH, 2012.

[86] S. Omoigui, “The interleukin-6 inflammation pathway from cholesterol to aging–role of statins, bisphosphonates and plant polyphenols in aging and age-related diseases,” *Immunity & Ageing*, vol. 4, no. 1, p. 1, 2007.

[87] J. B. Croft, J. L. Cresanta, L. S. Webber et al., “Cardiovascular risk in parents of children with extreme lipoprotein cholesterol levels: the Bogalusa Heart Study,” *Southern Medical Journal*, vol. 81, no. 3, pp. 341–9, 353, 1988.

[88] I. Jialal, “Evolving lipoprotein risk factors: lipoprotein(a) and oxidized low-density lipoprotein,” *Clinical Chemistry*, vol. 44, no. 8, pp. 1827–1832, 1998.

[89] S. E. Nissen, T. Tsunoda, E. M. Tuzcu et al., “Effect of recombinant ApoA-I Milano on coronary atherosclerosis in patients with acute coronary syndromes: a randomized controlled trial,” *Journal of the American Medical Association*, vol. 290, no. 17, pp. 2292–2300, 2003.

[90] G. Walldius, I. Jungner, A. H. Aastveit, I. Holme, C. D. Furberg, and A. D. Sniderman, “The apoB/apoA-I ratio is better than the cholesterol ratios to estimate the balance between plasma proatherogenic and antiatherogenic lipoproteins and to predict coronary risk,” *Clinical Chemistry and Laboratory Medicine*, vol. 42, no. 12, pp. 1355–1363, 2004.

[91] B. Singh, T. K. Bhat, and B. Singh, “Exploiting gastrointestinal microbes for livestock and industrial development - review -,” *Asian-Australasian Journal of Animal Sciences*, vol. 14, no. 4, pp. 567–586, 2001.

[92] Y. Xiong, N. Miyamoto, K. Shibata et al., “Short-chain fatty acids stimulate leptin production in adipocytes through the G protein-coupled receptor GPR41,” *Proceedings of the National Academy of Sciences of the United States of America*, vol. 101, no. 4, pp. 1045–1050, 2004.

[93] Y. T. Ahn, G. B. Kim, K. S. Lim, Y. J. Baek, and H. U. Kim, “Deconjugation of bile salts by *Lactobacillus acidophilus* isolates,” *International Dairy Journal*, vol. 13, no. 4, pp. 303–311, 2003.

[94] P. De Boever and W. Verstraete, “Bile salt deconjugation by lactobacillus plantarum 80 and its implication for bacterial toxicity,” *Journal of Applied Microbiology*, vol. 87, no. 3, pp. 345–352, 1999.

[95] N. I. Doncheva, G. P. Antov, E. B. Softova, and Y. P. Nyagolov, “Experimental and clinical study on the hypolipidemic and antisclerotic effect of Lactobacillus Bulgaricus strain GB N 1 (48),” *Nutrition Research*, vol. 22, no. 4, pp. 393–403, 2002.

[96] D. O. Noh and S. E. Gilliland, “Influence of bile on cellular integrity and *β* -galactosidase activity of *Lactobacillus acidophilus*,” *Journal of Dairy Science*, vol. 76, no. 5, pp. 1253–1259, 1993.

[97] M. L. Jones, C. J. Martoni, M. Parent, and S. Prakash, “Cholesterol-lowering efficacy of a microencapsulated bile salt hydrolase-active Lactobacillus reuteri NCIMB 30242 yoghurt formulation in hypercholesterolaemic adults,” *The British Journal of Nutrition*, vol. 107, no. 10, pp. 1505–1513, 2012.

[98] H. Kimoto, S. Ohmomo, and T. Okamoto, “Cholesterol removal from media by lactococci,” *Journal of Dairy Science*, vol. 85, no. 12, pp. 3182–3188, 2002.

[99] H. S. Lye, G. R. Rahmat-Ali, and M. T. Liong, “Mechanisms of cholesterol removal by lactobacilli under conditions that mimic the human gastrointestinal tract,” *International Dairy Journal*, vol. 20, no. 3, pp. 169–175, 2010.

[100] H. S. Lye, G. Rusul, and M. T. Liong, “Removal of cholesterol by lactobacilli via incorporation and conversion to coprostanol,” *Journal of Dairy Science*, vol. 93, no. 4, pp. 1383–1392, 2010.

[101] Y. R. Chiang, W. Ismail, D. Heintz, C. Schaeffer, A. Van Dorsselaer, and G. Fuchs, “Study of anoxic and oxic cholesterol metabolism by Sterolibacterium denitrificans,” *Journal of Bacteriology*, vol. 190, no. 3, pp. 905–914, 2008.

[102] M.-T. Liong, F. R. Dunshea, and N. P. Shah, “Effects of a synbiotic containing Lactobacillus acidophilus ATCC 4962 on plasma lipid profiles and morphology of erythrocytes in hypercholesterolaemic pigs on high- and low-fat diets,” *The British Journal of Nutrition*, vol. 98, no. 4, pp. 736–744, 2007.

[103] B. H. Arjmandi, J. Craig, S. Nathani, and R. D. Reeves, “Soluble dietary fiber and cholesterol influence in vivo hepatic and intestinal cholesterol biosynthesis in rats,” *The Journal of Nutrition*, vol. 122, no. 7, pp. 1559–1565, 1992.

[104] M. Kim and H. K. Shin, “The water-soluble extract of chicory influences serum and liver lipid concentrations, cecal shortchain fatty acid concentrations and fecal lipid excretion in rats,” *The Journal of Nutrition*, vol. 128, no. 10, pp. 1731–1736, 1998.

[105] C. L. Dikeman, M. R. Murphy, and G. C. Fahey Jr., “Dietary fibers affect viscosity of solutions and simulated human gastric and small intestinal digesta,” *The Journal of Nutrition*, vol. 136, no. 4, pp. 913–919, 2006.

[106] S. A. Moser and D. C. Savage, “Bile salt hydrolase activity and resistance to toxicity of conjugated bile salts are unrelated properties in lactobacilli,” *Applied and Environmental Microbiology*, vol. 67, pp. 3476–3480, 2001.

[107] S. Y. Lin, J. W. Ayres, W. Winkler Jr., and W. E. Sandine, “*Lactobacillus* effects on cholesterol: in vitro and in vivo results,” *Journal of Dairy Science*, vol. 72, no. 11, pp. 2885–2899, 1989.

[108] F. D. Gilliland, R. Mahler, W. C. Hunt, and S. M. Davis, “Preventive health care among rural American Indians in New Mexico,” *Preventive Medicine*, vol. 28, no. 2, pp. 194–202, 1999.

## Figures and Tables

**Figure 1:**
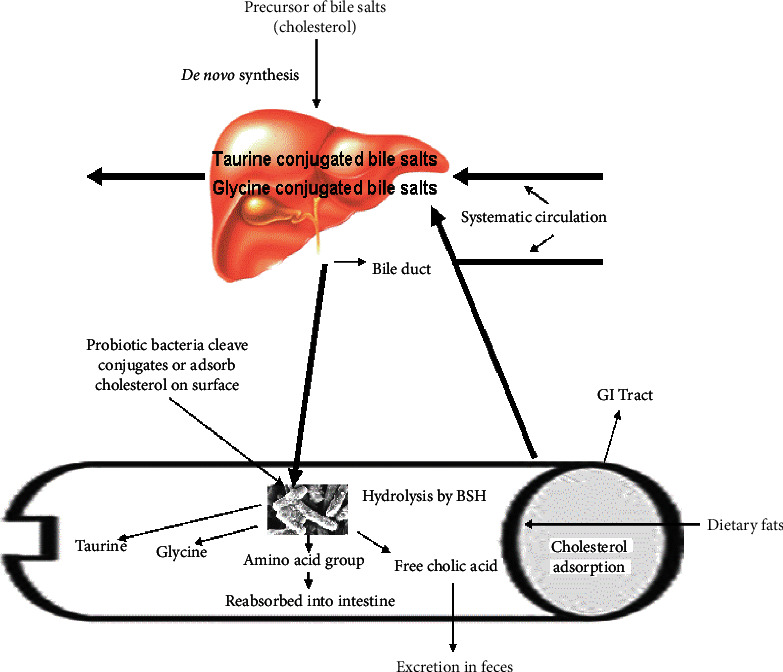
Illustration depicting cholesterol as a precursor for the synthesis of new bile acids and the cholesterol-lowering role of bile salt hydrolase (BSH).

**Figure 2:**
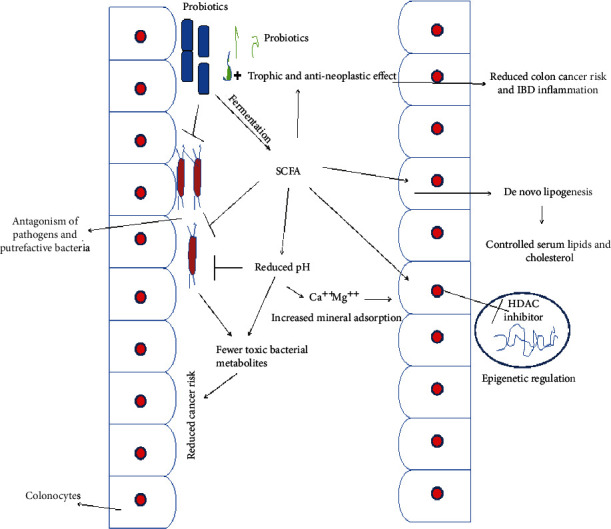
Role of probiotic's metabolites as epigenetic strategy for controlling hypercholesterolemia and colon cancers.

**Table 1:** List of some potential bacteria showing BSH activity.

Probiotic organisms with BSH activity	References
*Bifidobacterium adolescentis*	Tanaka et al. [49, 55]
*B. animalis*	Tanaka et al. [49, 55]
*B. breve*	Tanaka et al. [49]
*B. infantis*	Tanaka et al. [55]
*B. longum*	Tanaka et al. [49]
*Bifidobacterium* sp.	Grill et al. [62]
*Lactobacillus acidophilus*	Corzo and Gilliland [45, 46]
*L. casei*	Corzo and Gilliland [45, 46]
*L. gasseri*	Tanaka et al. [49, 55]
*L. helveticus*	Tanaka et al. [49, 55]
*L. paracasei* subsp. *paracasei*	Moser and Savage [106]
*L. rhamnosus*	Tanaka et al. [49]; Moser and Savage [106]
*L. plantarum*	De Smet et al. [8]

**Table 2:** Summary of major findings for probiotic mediated cholesterol reduction.

Probiotic organism	Experimental system	Major findings [reference]
Unknown (fermented milk)	Maasai tribesmen in Africa	Low serum cholesterol [72]
Unknown (yogurt)	Human subjects	Reduced serum cholesterol [73]
*L. acidophilus*	Culture media	Cholesterol removal, better survival in cholesterol medium [31]
*Bifidobacterium*	Culture media	Removal of cholesterol [25]
*L. acidophilus*	Culture media	Cholesterol assimilation [74]
Probiotic-fermented milk	Rats	Cholesterol reducing efficacy [75]
*L. reuteri*	Mice	Reduced blood cholesterol, decreased triglycerides [76]
*Bifidobacterium* milk	Rats, human	Reduced cholesterol, decreased triglyceride, decreased LDL, increased HDL [77]
Yoghurt containing *B. lactis or B. longum*	Rats	Reduced cholesterol, decreased triglyceride, decreased LDL, increased HDL [78]
*L. plantarum*	Culture media	Cholesterol assimilation [17]
*L. bulgaricus* and *L. acidophilus*	Humans	Decreased cholesterol [107]
*L. sporogenes*	Humans	Decreased cholesterol, reduced LDL cholesterol [79]
*L. acidophilus*	Humans	Decreased cholesterol [108]
*E. faecium*	Humans	Decreased cholesterol, decreased triglyceride, decreased LDL, increased HDL [81]
Microencapsulated BSH-active *L. reuteri* NCIMB 30242	Humans	Decreased total cholesterol, reduced LDL cholesterol, decreased apoB-100, decreased non-HDL cholesterol [97]
